# Mating-type genes of the anamorphic fungus *Ulocladium botrytis* affect both asexual sporulation and sexual reproduction

**DOI:** 10.1038/s41598-017-08471-3

**Published:** 2017-08-11

**Authors:** Qun Wang, Shi Wang, Chen Lin Xiong, Timothy Y. James, Xiu Guo Zhang

**Affiliations:** 1 Department of Plant Pathology, Shandong Provincial Key Laboratory for Biology of Vegetable Diseases and Insect Pests, Shandong Agricultural University, 61, Daizong Street, Tai’an, Shandong 271018 China; 2 Department of Evolutionary Biology, University of Michigan, Ann Arbor, MI 48109 USA

## Abstract

*Ulocladium* was thought to be a strictly asexual genus of filamentous fungi. However, *Ulocladium* strains were shown to possess both *MAT1-1-1* and *MAT1-*2*-1* genes as observed in homothallic filamentous Ascomycetes. Here, we demonstrate that the *U. botrytis MAT* genes play essential roles for controlling asexual traits (conidial size and number). Using reciprocal genetic transformation, we demonstrate that *MAT* genes from the related heterothallic species *Cochliobolus heterostrophus* can also influence *U. botrytis* colony growth, conidial number and size, and have a strong effect on the range of the number of septa/conidium. Moreover, *U. botrytis MAT* genes can also affect similar aspects of asexual reproduction when expressed in *C. heterostrophus*. Heterologous complementation using *C. heterostrophus MAT* genes shows that they have lost the ability to regulate sexual reproduction in *U. botrytis*, under the conditions we employed, while the reciprocal heterologous complementation demonstrates that *U. botrytis MAT* genes have the ability to partially induce sexual reproduction in *C. heterostrophus*. Thus, the genetic backgrounds of *C. heterostrophus* and *U. botrytis* play significant roles in determining the function of *MAT* genes on sexual reproduction in these two fungi species. These data further support the role of *MAT* genes in controlling asexual growth in filamentous Ascomycetes but also confirm that heterothallic and homothallic Dothideomycete fungi can be interconverted by the exchange of *MAT* genes.

## Introduction

Sexual reproduction in filamentous ascomycetes is controlled by a single regulatory mating-type locus referred to as the mating-type locus or *MAT* ^1, 2^. The mating-type locus consists of two dissimilar DNA sequences in the mating partners, termed *MAT1-1* and *MAT1-2* idiomorphs^3^. *MAT1-1* encodes a protein with an alpha-box (α-box) DNA-binding domain, whereas *MAT1-2* encodes a protein with an HMG-box (high mobility group) DNA-binding motif. The α-box or HMG-box domain proteins specify two alternative transcription factors that permit each mating type to induce specific expression of many other genes required during and after mating, in particular, the genes that regulate pheromone precursors and pheromone receptors that are essential for cells of opposite mating types to attract each other and cause fertilization^2, 4–6^.

Mating behavior in filamentous ascomycetes can be either homothallic (self-fertile) or heterothallic (self-sterile) in the same genus^1^. Initiation of the sexual cycle is the step that mainly distinguishes heterothallic and homothallic species. The heterothallic species of filamentous ascomycetes are known to possess either one or the other idiomorph at the *MAT1* locus. In contrast to the heterothallic species, homothallic species carry both MAT idiomorphs in a single genome, usually closely linked or fused^2, 7^. Over the past decade, mating-type genes have been identified and characterized in an increasing number of filamentous ascomycetes, where their function as master regulators of sexual reproduction has been conserved^7–16^. However, for approximately half of all filamentous ascomycetes species there is no known sexual state^17^. Presently, the question is whether these fungi, which only reproduce in a vegetative state, have abandoned sexual reproduction altogether. Alternatively, their sexual states could be small, inconspicuous, or only initiated under unusual conditions. Evidence suggests, following molecular investigation, that even the many putatively asexual filamentous ascomycetes species have genomes with *MAT* genes^18^, some of which are constitutively transcribed, providing appropriate evidence for sexual potential that is morphologically absent^19–25^. Therefore, it is of great interest to determine whether the occurrence of *MAT* genes in an asexual species is a sign of realized mating, a relictual unused gene set, or a pathway that evolved to regulate another function.

Traditionally, the only way to determine whether any filamentous ascomycete species can reproduce sexually is by observation of their reproductive characteristics. Currently, the recent breakthroughs in the understanding of mating in ascomycetes following the cloning of mating-type genes in combination with genomics has made it possible to answer questions about the role of *MAT* genes in presumably asexual fungi. First, since the primary function of *MAT* genes is regulatory control of sex, their presence in asexual fungi can be presumed a necessary condition for sex to occur. Second, it can be assessed whether the *MAT* genes are properly expressed under controlled conditions and developmentally regulated in a manner consistent with sexual reproduction. Last, it can be tested whether mutations in *MAT* genes in asexual species occur more frequently and unpredictably than mutations in sexual species as a process of accumulation of mutations in the unused or neo-functionalized *MAT* genes.

The *MAT* genes have been well-studied in putatively asexual ascomycete species^24, 26–28^. *Ulocladium* is genus of ascomycetes closely allied with the anamorphic (asexual) genera *Alternaria*, *Embellisia*, *Nimbya* and *Stemphylium* in the order Pleosporales (Dothideomycetes)^28^. *Ulocladium* contains more than 29 species^29–33^ and is closely allied with *Alternaria* and *Stemphylium*. Teleomorphs are known from several species in these two allied genera; their sexual states patterns are *Alternaria*/*Lewia* and *Stemphylium*/*Pleospora*, respectively^34, 35^. However, no sexual state has yet been identified for *Ulocladium*. *Ulocladium* is therefore thought to be strictly asexual. In addition, most species within these five genera are only allied to asexual states. *Alternaria* is considered to be a largely asexual genus because most of the members have no known teleomorph yet are still known to carry expressed *MAT* genes in a heterothallic arrangement^19, 24^. The genus *Stemphylium* is the anamorphic stage of the teleomorph *Pleospora* ^36–38^. Some *MAT* loci of the *Stemphylium* species contain a single idiomorph (self-sterile), either *MAT1-1* or *MAT1-2*, whereas others contain a unique fusion of *MAT1-1* and *MAT1-2* regions (self-fertile)^2, 39^. However, the sexual state has not been identified in most species of *Stemphylium*. The *MAT* locus organization is unknown for most members of the genus *Alternaria* ^39^.

Previously, we identified the full-length sequences of *MAT1-1-1* and *MAT1-2-1* genes for 26 *Ulocladium* species. Notably, both *MAT1-1-1* and *MAT1-2-1* genes were detected in the same haploid genome of all 26 *Ulocladium* species which appear to similar to that of *Ophiocordyceps sinensis* ^40^, and thus all of the *Ulocladium* species have the potential to be homothallic^30^. Transcriptional analysis on the basis of qRT-PCR showed that both *MAT1-1-1* and *MAT1-2-1* genes were expressed and may be functional in all 26 *Ulocladium* species, suggesting that all these *Ulocladium* species might have the potential to reproduce sexually^30^.

In this study, we focused on the type species *U. botrytis* of *Ulocladium* ^29^ and addressed the question of whether *U. botrytis MAT1-1-1* or *MAT1-2-1* genes lost the ability for sexual reproduction using genetic disruption and heterologous expression. In addition, we tested whether *MAT* genes influence asexual reproduction of *Ulocladium* species under natural conditions. Here, we first demonstrated that *U. botrytis MAT1-1-1* and *MAT1-2-1* play essential roles in colony growth and conidial size and number in *U. botrytis* using both separate *MAT1*-*1-1* or *MAT1-2-1* deletions and double deletions. Then, using heterologous expression, we showed that mating-type genes, regardless of whether they come from a heterothallic fungus (*C. heterostrophus*) or the anamorphic fungus (*U. botrytis*), regulate the expression of only asexual reproduction in the anamorphic fungus, whereas *MAT* genes from both the asexual and sexual species are capable of inducing sexual development when tested in the sexual species. This study provides insights into the functional role of *MAT* genes in asexual filamentous fungi where sexual reproduction is rare or absent and provides additional evidence that *MAT* genes may regulate important processes not directly related to sexual reproduction, i.e., asexual sporulation.

## Results

### Influence of *U. botrytis MAT1-1-1* and *MAT1-2-1* on vegetative growth and asexual sporulation

**Figure 1:**
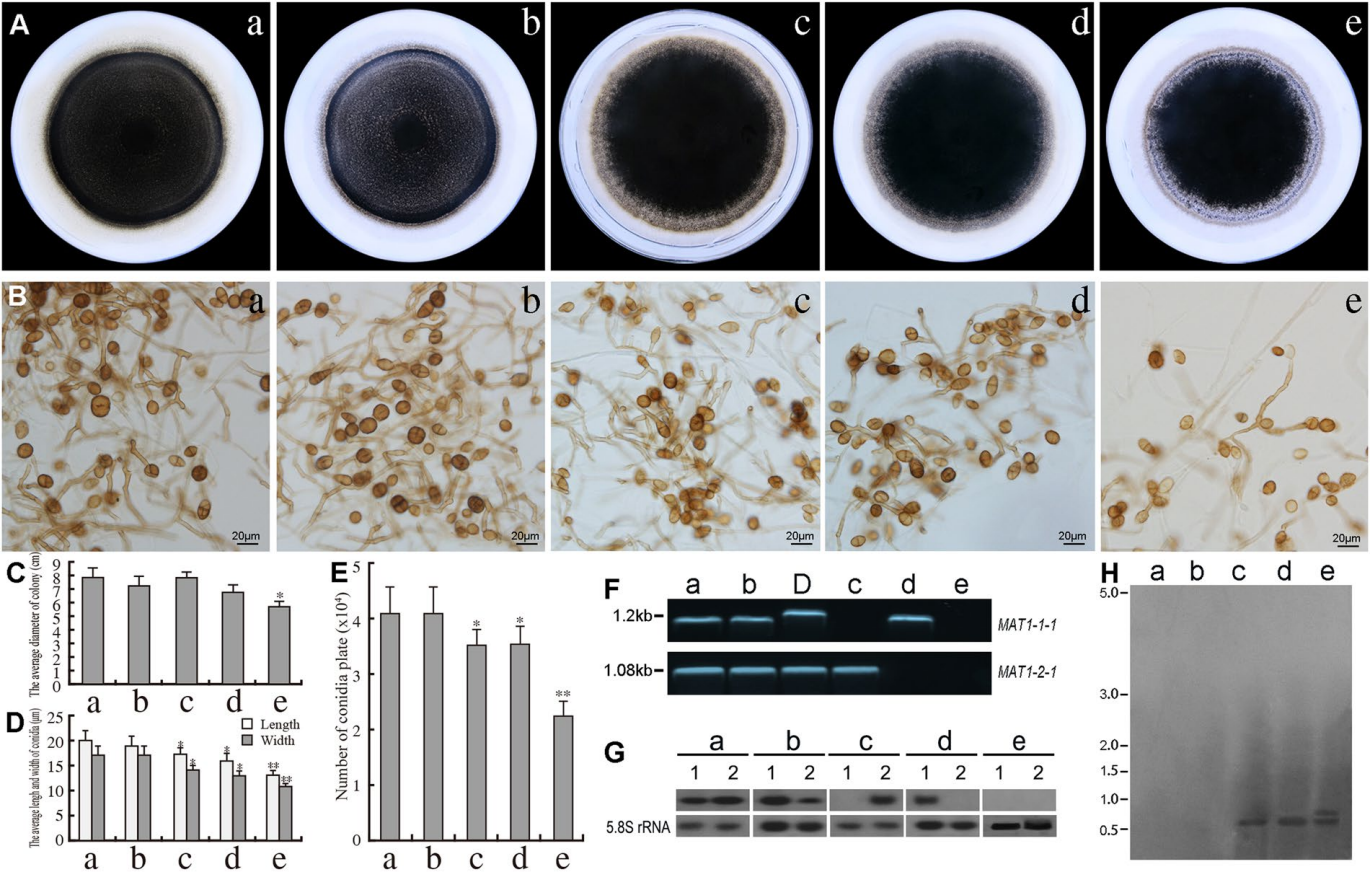
Effect of deletion of the *U. botrytis MAT* genes on colony morphology, conidial size and number. (**A**,**C**) Growth and diameter of the colonies of different mutants at 12 days after incubation. Colony growth rates were determined from at least 25 plates. (**B**,**D**) Variation in conidial size. **L** = Length, **W** = Width. The average size of conidia was determined from at least 50 conidia. Photographs were taken 12 days after incubation. (**B**,**E**) Variation in conidial number. The number of conidia produced per plate from cultures grown on PCA plates for 12 days under standard conditions. Error bars represent standard errors calculated using three replicates for each sample. ‘*’ indicates a significant difference from WT (*P* < 0.05) using Student’s *t*-*test*. ‘**’ indicates a significant difference from WT (*P* < 0.01) using Student’s *t*-*test*. (**F**) PCR analysis of the transcription of the *MAT* genes in different deletion lines. D-DNA template of WT. (**G**) Northern blot analysis. Twenty micrograms of total RNA, isolated from WT, CK, and all mutant strains, were loaded per lane. The Northern blot was probed using *MAT1-1-1* and *MAT1-2-1* gene-specific probes. A 5.8S rRNA-specific probe was used as positive control. (**H**) For Southern blot analysis, both *hygB* and G418 specific probes were used to detect transgene insertion. WT and CK have no *hygB* and G418 specific insertion. a WT (Wild-type *U. botrytis*). b CK is an empty vector transformant. c *ΔmatUbMAT-1*, G418 was used to detect transgene insertion. d. *ΔmatUbMAT-2, hygB* was used to detect transgene insertion. e *DmUbMAT*-*1*, *hygB* and G418 were individually used to detect transgene insertion. Each experiment was repeated at least three independently times.

We have previously cloned and described the structural organization of *MAT1-1-1* and *MAT1-2-1* from the asexual species *U. botrytis* ^30^. To test the functions of *MAT1-1-1* and *MAT1-2-1* genes in *U. botrytis*, we created *MAT1-1-1* or *MAT1-2-1*, and *MAT1-1-1*/*1-2-1* deletion mutants using the split-marker method. The single gene deletion mutants *ΔmatUbMAT*-*1* and *ΔmatUbMAT-2* and double mutants *DmUbMAT-1* shown in Table S1 were confirmed by PCR (Fig. 1F), Northern blot (Fig. 1G) and Southern blot assays (Fig. 1H). The colony diameters of *ΔmatUbMAT-1* and *ΔmatUbMAT-2* (Fig. 1A,C: c,d) were very similar to those of WT and CK (Empty vector transformant) (Fig. 1A,C: a,b), whereas the colony diameters of *DmUbMAT-1* (Fig. 1A,C: e) were slightly smaller than those of the two controls and either of the two single mutants (*P* < 0.05). The colony borders of these three mutants (Fig. 1A: c,d,e) were loose in contrast to WT and CK (Fig. 1A: a,b), and *DmUbMAT-1* showed significant incompactness around the colony borders (Fig. 1A: e). *MAT* expression levels influenced the size of the conidia (Fig. 1B,D), and the conidial sizes of *ΔmatUbMAT-1* (17 × 14 μm^2^) and *ΔmatUbMAT-2* (16 × 13 μm^2^) were slightly smaller than those of WT (20 × 17 μm^2^) and CK (19 × 16 μm^2^) (*P* < 0.05). The conidial sizes of *DmUbMAT-1* (13 × 11 μm^2^) were significantly smaller than those of conidia formed by WT, CK and either of the two single mutants (*P* < 0.01). *MAT* expression levels also were correlated with the total number of conidia produced by three different mutants compared with two controls (Fig. 1B,E). The two single mutants *ΔmatUbMAT-1* and *ΔmatUbMAT-2* produced fewer conidia than did WT and CK (*P* < 0.05; Wilcoxon rank-sum *test*), but *DmUbMAT-1* produced the least conidia. These data demonstrate that *MAT1-1-1* and *MAT1-2-1* play roles in both colony growth and conidial size and number in *U. botrytis*.

### Heterothallic *C. heterostrophus MAT1-1-1* and *MAT1-2-1* also influence vegetative growth and asexual sporulation in *U. botrytis*

**Figure 2:**
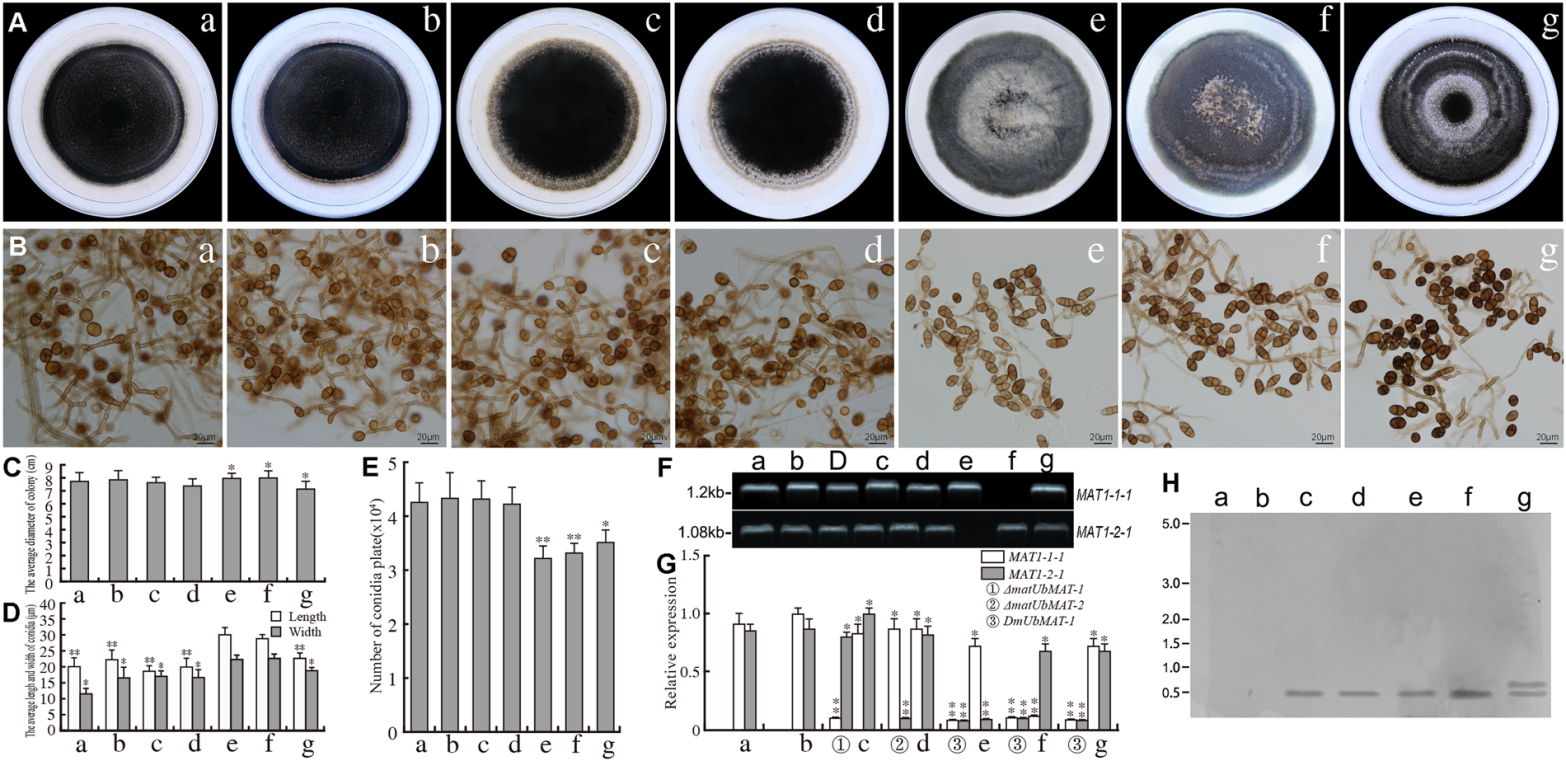
Effect of heterologous expression of *C. heterostrophus MAT* genes on asexual morphology in *U. botrytis* strains of *MAT* deletion lines. (**A**,**C**) Growth and diameter of colonies across the different transgenic lines at 12 days after incubation. Colony growth rates were determined from at least 25 plates. (**B**,**D**) Variation in conidial size. L = Length, W = Width. The average size of conidia were determined from at least 50 conidia. Photographs were taken at 12 days after incubation. (**B**,**E**) Variation in conidial number. Number of conidia produced per plate from cultures grown on PCA plates for 12 days under standard conditions. (**F**) PCR analysis of *MAT* gene transcription in different transgenic lines. D-DNA template of WT. (**G**) qRT-PCR analysis of mRNA expression levels of *MAT1-1-1* and *MAT1-2-1* in individual heterologous transgenic lines, relative to the constitutive control genes. WT and CK were used as negative controls. *Actin* gene was used as the reference gene. Error bars represent standard errors calculated using three biological replicates for each sample. ‘*’ indicates a significant difference from WT (*P* < 0.05) using a Student’s *t*-*test*. ‘**’ indicates a significant difference from WT (*P* < 0.01) using a Student’s *t-test*. (**H**) For Southern blot analysis, both *hygB* and G418 specific probes were used to detect transgene insertion as shown in Table S1. WT and CK have no *hygB* and G418 specific insertion. a WT (Wild-type *U. botrytis*), b CK is an empty vector transformant. c *ΔmatUbMAT-1*{*ChMAT*}*-1*, d. *ΔmatUbMAT-2*{*ChMAT*}*-1*, e *DmUbMAT-1*{*ChMAT*}*-1-1*, f. *DmUbMAT-1*{*ChMAT*}*-2-1*, g *DmUbMAT-1*{*ChMAT*}*-3-1*. Each experiment was repeated at least three times.

To test the functions of the *C. heterostrophus MAT1-1-1* and *MAT1-2-1* in the stable *U. botrytis* deletion mutants *ΔmatUbMAT-1*, *ΔmatUbMAT-2*, and *DmUbMAT-1*, we created transformants *ΔmatUbMAT-1*{*ChMAT*}, *ΔmatUbMAT-2*{*ChMAT*}, *DmUbMAT-1*{*ChMAT*}*-1*, *DmUbMAT-1*{*ChMAT*}*-2*, and *DmUbMAT-1*{*ChMAT*}*-*3 using previously described methods. Each of the *U. botrytis MAT* deletion mutants was transformed with the corresponding gene from *C. heterostrophus*, i.e., *ΔmatUbMAT-1*{*ChMAT*} contains the *MAT1-1-1* gene of C*. heterostrophus*. Each of these transgenes conferred to the *MAT* deletion mutants of *U. botrytis* the same phenotypes of colony growth, conidial number and size, and compartmentalization (data not shown). Thus, we only analyzed the five typical transformants *ΔmatUbMAT-1*{*ChMAT*}*-1*, *ΔmatUbMAT-*2{*ChMAT*}*-1*, *DmUbMAT-1*{*ChMAT*}*-1-1*, *DmUbMAT*-1{*ChMAT*}*-2-1*, and *DmUbMAT-1*{*ChMAT*}*-3-1* in subsequent experiments (Table S1). The PCR, Southern blot, and qRT-PCR analyses of these five typical transformants are shown in Fig. 2F,G,H.

The colony diameters of these five transformants (Fig. 2A,C: c,d,e,f,g) were very similar to WT and CK (Fig. 2A,C: a,b). The colony borders of *ΔmatUbMAT-1*{*ChMAT*}*-1* and *ΔmatUbMAT-2*{*ChMAT*}*-1* (Fig. 2A: c,d) are incompact in contrast to WT and CK (Fig. 2A: a,b) and three other typical transformants in the *DmUbMAT-1* background (Fig. 2A: e,f,g). Notably, no significant differences in the colony borders between *ΔmatUbMAT-1*{*ChMAT*}*-1* and *ΔmatUbMAT*-*2*{*ChMAT*}-*1* (Fig. 2A: c,d) and the two single mutants *ΔmatUbMAT-1* and *ΔmatUbMAT-2* (Fig. 1A: c,d) were found under standard conditions. As shown in Fig. 2A: e,f,g, the cultures of these three *DmUbMAT-1* transformants became loose and ringed in a slight gray color while the single gene deletion background transformants (Fig. 2A: c,d) and either of the two controls (Fig. 2A: a,b) were very compact and pigmented in a constant dark color.

*C. heterostrophus MAT* gene heterologous expression in asexual *U. botrytis* can affect the variation of conidial sizes and number in these different transformants. As shown in Fig. 2B,D,E (c,d), the conidial sizes and numbers of the *ΔmatUbMAT-1*{*ChMAT*}*-1* and *ΔmatUbMAT-2* {*ChMAT*}*-1* were very similar to those of WT and CK (Fig. 2B,D,E: a,b). The conidial sizes of *DmUbMAT-1*{*ChMAT*}*-1-1* (30 × 22 μm^2^) and *DmUbMAT-*1{*ChMAT*}*-2-1* (29 × 23 μm^2^) (Fig. 2B,D: e,f) were significantly larger than those of WT (20 × 17 μm^2^) and CK (22 × 16 μm^2^) (Fig. 2B,D: a,b) and of the two other transformants *ΔmatUbMAT-1*{*ChMAT*}*-1* (19 × 17 μm^2^) and *ΔmatUbMAT-*2 {*ChMAT*}*-1* (20 × 16 m^2^) (*P* < 0.01) (Fig. 2B,D: c,d). The conidial sizes of the *DmUbMAT-1*{*ChMAT*}*-3-1* (23 × 19 μm^2^) (Fig. 2B,D: g) were slightly larger than those of the two controls and *ΔmatUbMAT-1*{*ChMAT*}*-1* and *ΔmatUbMAT*-2 {*ChMAT*}*-1* (*P* < 0.05) (Fig. 2B,D: a,b,c,d) but were also significantly smaller than those of *DmUbMAT-1*{*ChMAT*}*-1-1* and *DmUbMAT-*1{*ChMAT*}*-2-1* (*P* < 0.01) (Fig. 2B,D: e,f). On the other hand, the number of conidia produced by *DmUbMAT-1*{*ChMAT*}*-1-1*, *DmUbMAT-1*{*ChMAT*}*-2-1*, and *DmUbMAT-1*{*ChMAT*}*-3-1* (Fig. 2B,E: e,f,g) were significantly fewer than those of WT, CK, and of the two single gene deletion backgrounds with transgenes (Fig. 2B,E: a,b,c,d) (*P* < 0.01). The range in the number of septa/conidium was 0-1 within CK, WT, *ΔmatUbMAT-1*{*ChMAT*}*-1* and *ΔmatUbMAT-*2{*ChMAT*}*-1* (Fig. 2B: a,b,c,d), whereas *DmUbMAT-1*{*ChMAT*}*-1-1* and *DmUbMAT-1*{*ChMAT*}*-2-1* had 1-4 septa/conidium and most had 2-3 (Fig. 2B: e,f). However, the mature conidia of *DmUbMAT-1*{*ChMAT*}-*3-1* (Fig. 2B: g) was restored to 0-1 septa/conidium as in the WT and CK and became more darkly pigmented and distinctly different from the two controls and each of the four other transformants (Fig. 2B: a,b,c,d,e,f). These results indicated that the *C. heterostrophus MAT1-1-1* and *MAT1-2-1* transgenes could regulate similar asexual reproduction traits as observed for *U. botrytis MAT* genes.

### Expression of *U. botrytis MAT1-1-1* and *MAT1-2-1* in *C. heterostrophus* influences vegetative growth and asexual sporulation

**Figure 3:**
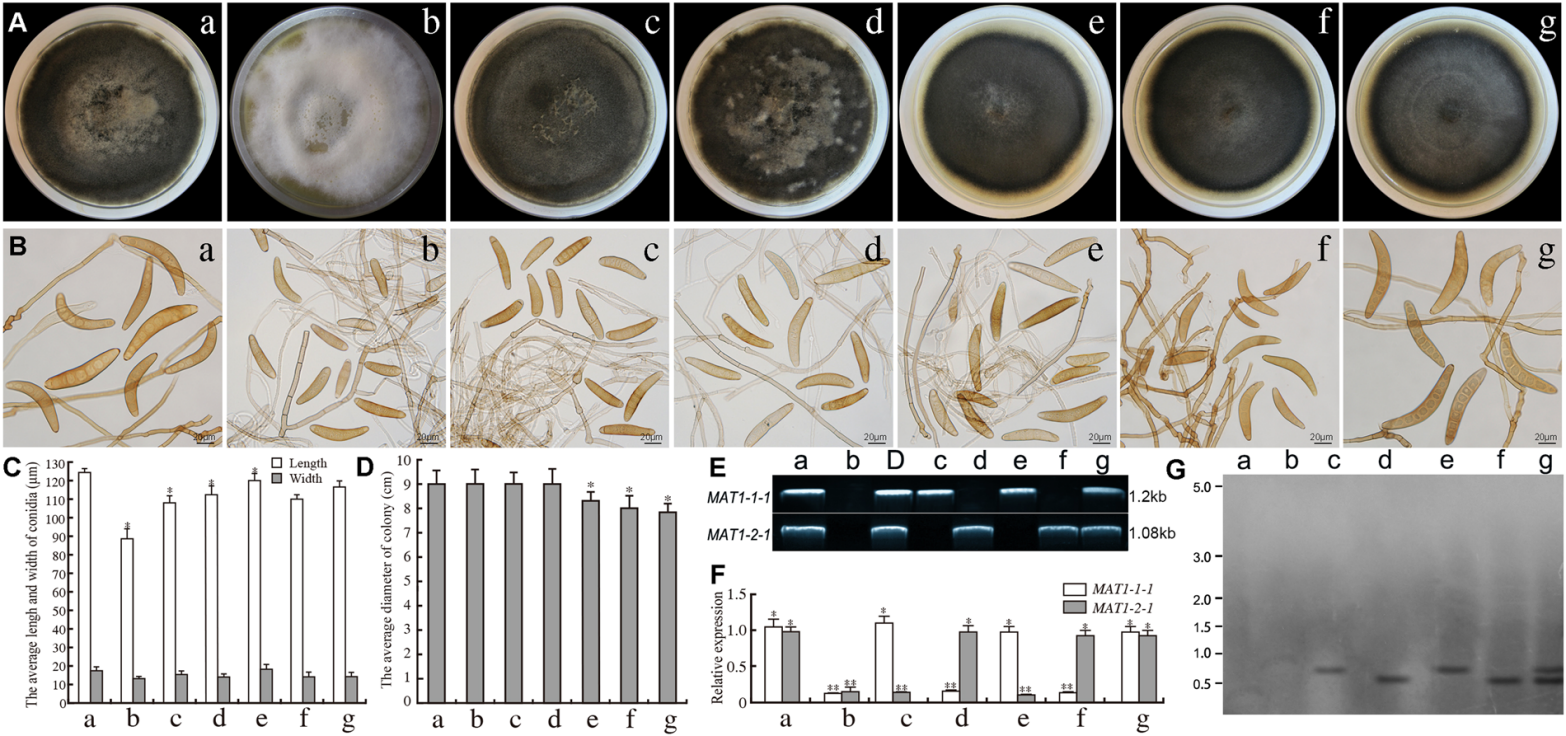
Effect of transformation of *U. botrytis MAT* genes on asexual morphology of *C. heterostrophus* C4-41.7 (MAT-0). (**A**,**D**) Growth and diameter of the colonies of the different strains at 12 days after incubation. Colony growth rates were determined from at least 25 plates. (**B**,**C**) Variation in conidial size. **L** = Length, **W** = Width. The average size of conidia was determined from at least 50 conidia. Photographs were taken 12 days after incubation. (**E**) RT-PCR analysis of the transcription of *MAT* genes in different transgenic lines. D-DNA template of WT. (**F**) qRT-PCR analysis of mRNA expression levels of *MAT1-1-1* and *MAT1-2-1* in individual heterologous transgenic lines as described above, relative to the constitutive control lines. *C. heterostrophus* strains 2847, C4-41.7 (MAT_0_), 2829 and 2849 were used as negative controls. *Actin* was used as a positive control. Error bars represent standard errors calculated using three biological replicates for each sample. ‘*’ indicates a significant difference from WT (*P* < 0.05) using a Student’s *t*-*test*. ‘**’ indicates a significant difference from WT (*P* < 0.01) using a Student’s *t-test*. (**G**) For Southern blot analysis, both *hygB* and G418 specific probes were used to detect transgene insertion as shown in Table S1. WT and WT1 have no *hygB* and G418 specific insertion. a WT is *C. heterostrophus* (2847). b WT1 is *C. heterostrophus* C4-41.7 (MAT-0). c WT2 is *C. heterostrophus* C5 (2829). d WT3 is *C. heterostrophus* C4 (2849). e *ChΔmat0* {*UbMAT*}*-2*. f *ChΔmat0* {*UbMAT*}*-3*. g *ChΔmat0* {*UbMAT*}*-4*. Each experiment was repeated at least three times.

To determine whether *U. botrytis MAT1-1-1* and *MAT1-2-1* are involved in controlling colony growth and size and number of conidia in *C. heterostrophus*, three transformants were created and were used for subsequent analyses, including *ChΔmat0*{*UbMAT*}-2, *ChΔmat0*{*UbMAT*}-3 and *ChΔmat0* {*UbMAT*}-4 (Table S1). The genetic composition of these three transformants were confirmed by PCR, Southern blot and qRT-PCR (Fig. 3E,F,G: e,f,g) and compared to *C. heterostrophus* (2847), *C. heterostrophus* C4-41.7 (MAT-0), *C. heterostrophus* C5 (2829) and *C. heterostrophus* C4 (2849), which served as controls (Fig. 3E,F,G: a,b,c,d). The cultures of the three heterologous transformants (Fig. 3A,D: e,f,g) were often very compact and darkly pigmented in a constant manner with slightly small diameters compared with each of the four controls (Fig. 3A,D: a,b,c,d). The conidial sizes of *ChΔmat0* {*UbMAT*}*-2* (110 × 15 μm^2^) and *ChΔmat0* {*UbMAT*}*-3* (117 × 14 μm^2^) (Fig. 3C: e,f) were nearly the same as those of *C. heterostrophus* C5 (108 × 16 μm^2^) and *C. heterostrophus* C4 (112 × 14 μm^2^) (Fig. 3C: c,d). The conidia produced by *C. heterostrophus* C4-41.7 were the smallest in size (89 × 12 μm^2^) of all the untransformed strains (Fig. 3C: b). However, the conidial sizes of *ChΔmat0* {*UbMAT*}*-*4 (124 × 17 μm^2^) (Fig. 3C: g) were the largest and most similar to that of *C. heterostrophus* (2847) (120 × 19 μm^2^) (Fig. 3C: a). No clear differences in the number of conidia produced by these three transgenic strains and four controls were found under standard conditions (data not shown). For *C. heterostrophus* C4-41.7, the range in number of septa/conidium was 1–6, with a mean of 3–4 (Fig. 3B: b). Notably, *ChΔmat0*{*UbMAT*}*-2*, *ChΔmat0*{*UbMAT*}*-3*, and *C. heterostrophus* C5/C4 had 3–9 septa/conidium and most had 5-7 (Fig. 3B: c,d,e,f). In addition, *ChΔmat0*{*UbMAT*}*-*4 was nearly restored to the wild type strain *C. heterostrophus* (2847) that had 7–12 septa/conidium and most had 7–9 (Fig. 3B: a,g). Therefore, we concluded that the *U. botrytis MAT1-1-1* and *MAT1-2-1* genes could also affect asexual reproduction in *C. heterostrophus*.

### Effect of *C. heterostrophus MAT1-1-1* and *MAT1-2-1* genes on sexual reproduction in the anamorphic *U. botrytis*

**Figure 4:**
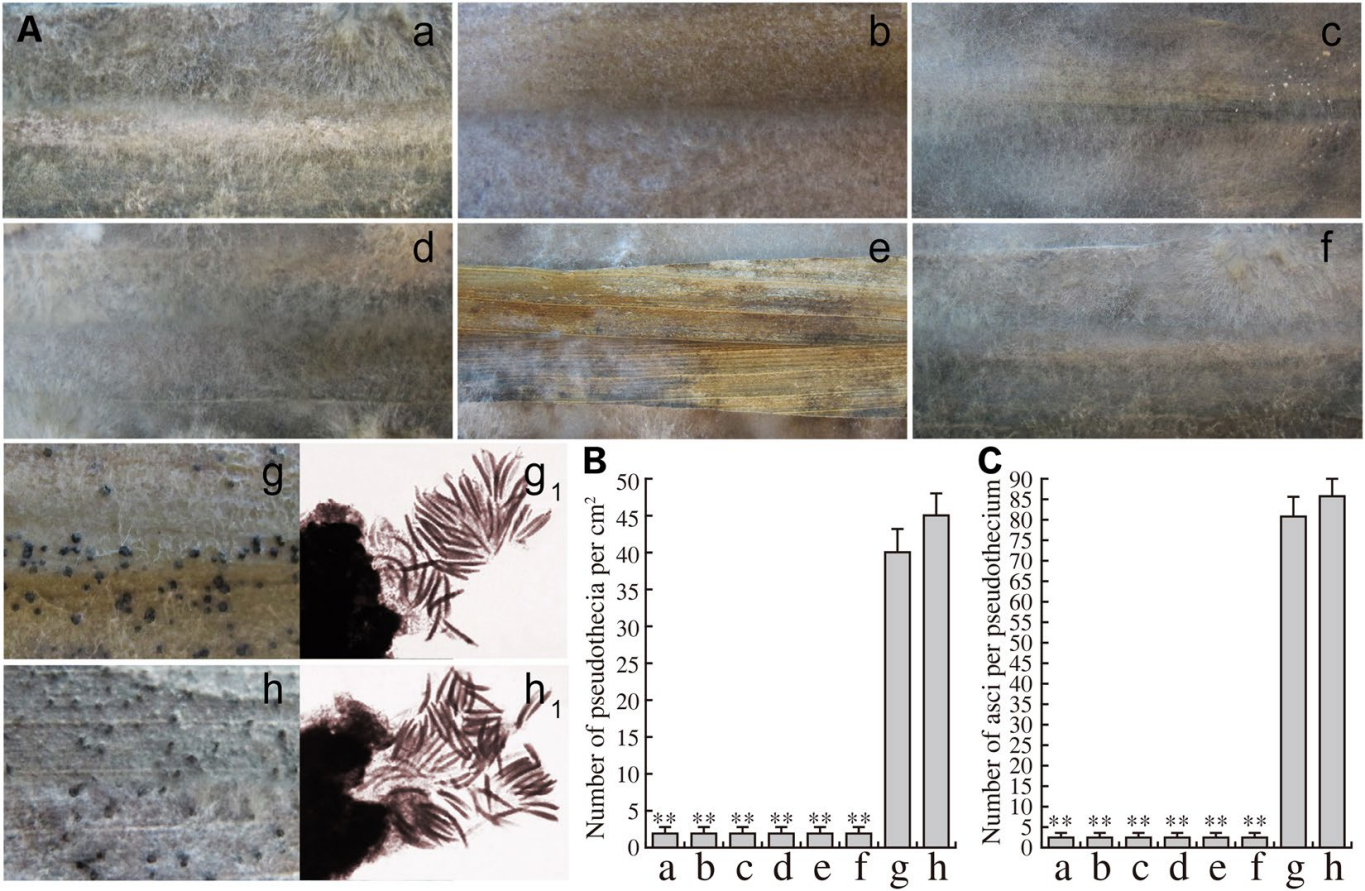
Effect of transformation of *C. heterostrophus MAT* genes on pseudothecia and asci formation in *U. botrytis*. (**A**) Pseudothecia formation was tested in different crosses or self matings on the surface of corn leaf substrates. (**B**) Average number of pseudothecia per square centimeter on the surface of the corn leaf. Error bars indicate 95% confidence intervals. No significant differences were observed in the number of pseudothecia between W1 and W2 (*P* > 0.05). (**C**) Average number of asci per pseudothecium. At least 10 pigmented pseudothecia were opened and the number of asci in each pseudothecium were recorded. Error bars indicate 95% confidence intervals. No significant differences were observed in the number of asci per pseudothecium between **WT1** and **WT2** (*P* > 0.05). a Cross-mating pattern *DmUbMAT-1*{*ChMAT*}*-1-1*× *DmUbMAT-1*{*ChMAT*}*-2-1*. b Self-mating pattern *DmUbMAT-1*{*ChMAT*}*-3-1*. c Self-mating pattern *ΔmatUbMAT-1*{*ChMAT*}*-1*. d Self-mating pattern *ΔmatUbMAT-2*{*ChMAT*}*-1*. e Cross-mating pattern *ΔmatUbMAT-1*× *ΔmatUbMAT-2*. f Self-mating pattern *U. botrytis* strain. All these crosses or self matings were completely sterile–no pseudothecia and asci were produced on the surface of corn leaf substrates. g and g_1_ Cross mating of *C. heterostrophus* C5× *C. heterostrophus* C4 (W1). h and h_1_ Self mating of *C. heterostrophus* (2847) (W2). Following W1 and W2 crosses, pseudothecia and asci were produced on the surface of corn leaf substrates.

To confirm whether a mating phenotype of the asexual *U. botrytis* was conferred by *C. heterostrophus MAT* transgenes, we conducted cross mating using *DmUbMAT-1* {*ChMAT*}*-1-1*× *DmUbMAT-1*{*ChMAT*}*-2-1* strains that carried compatible *C. heterostrophus MAT* genes and three tests of self-fertilization of strains with gene combinations expected to confer self-compatibility, including *DmUbMAT-1*{*ChMAT*}*-3-1*, *ΔmatUbMAT-1*{*ChMAT*}*-1*, and *ΔmatUbMAT-2*{*ChMAT*}*-1* (Table S2). The *DmUbMAT-1*{*ChMAT*}*-3-1* strain was transformed with both the *C. heterostrophus MAT1-1-1* and *MAT1-2-1* genes. The *ΔmatUbMAT-1*{*ChMAT*}*-1* strain contained *U. botrytis MAT1-2-1* transformed with *C. heterostrophus MAT1-1-1*. The *ΔmatUbMAT-2*{*ChMAT*}*-1* strain contained *U. botrytis MAT1-1-1* transformed with *C. heterostrophus MAT1-2-1*. *DmUbMAT-1*{*ChMAT*}*-1-1*× *DmUbMAT-1*{*ChMAT*}*-2-1* did not produce pseudothecia or asci after incubating on the surface of corn leaf substrates (Fig. 4A: a, Table S2), and these results were consistent with the three self matings which were also sterile (Fig. 4A: b,c,d, Table S2). Moreover, cross mating of *ΔmatUbMAT-1*× *ΔmatUbMAT-2* (Fig. 4A: e, Table S2) and self-mating of *U. botrytis* (Fig. 4A: f, Table S2) did not produce pigmented pseudothecia and asci on the surface of corn leaf substrates. In contrast, the number of pseudothecia per square centimeter and the number of asci per pseudothecium were much greater in cross matings of *C. heterostrophus* C5 × *C. heterostrophus* C4 (W1) (Fig. 4A: g and g_1_, Fig. 4B,C: g, Table S2) and were indistinguishable from those produced in self mating of *C. heterostrophus* (W2) (Fig. 4A: h and h_1_, Fig. 4B,C: h, Table S2). These data demonstrate that although the *C. heterostrophus MAT* genes are expressed in the *U. botrytis* transgenic strains (Fig. 2F,G), the *C. heterostrophus MAT* genes can not regulate sexual reproduction in the genetic background of the anamorphic *U. botrytis* strains.

### Effect of *U. botrytis MAT1-1-1* and *MAT1-2-1* genes on sexual reproduction in *C. heterostrophus*

**Figure 5:**
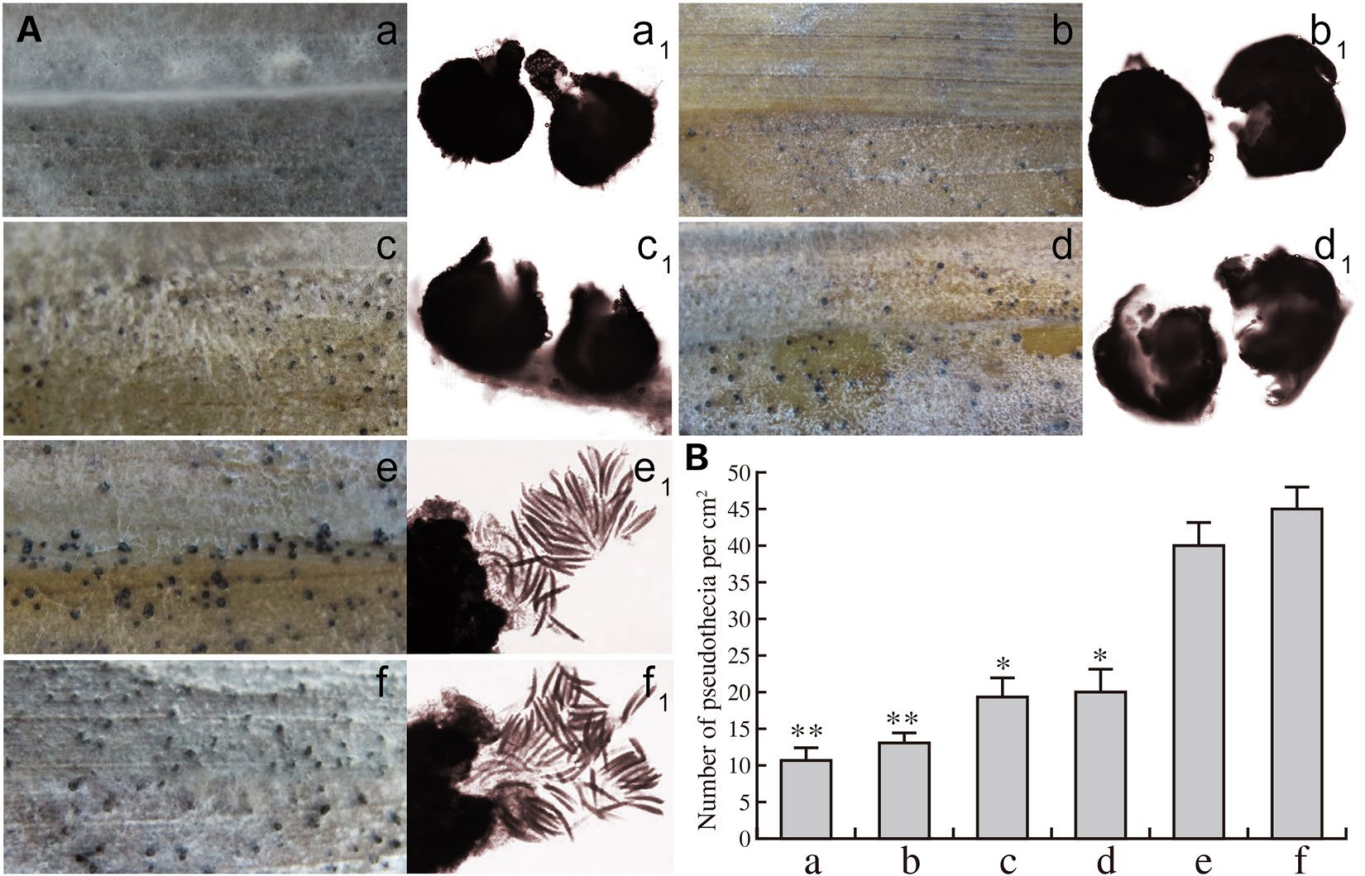
Effect of transformation of *U. botrytis MAT* genes on pseudothecia and asci formation in *C. heterostrophus*. (**A**) Pseudothecia formation in different cross or self matings on the surface of corn leaf substrates. (**B**) Average number of pseudothecia per square centimeter on the area of the corn leaf. Error bars indicate 95% confidence intervals. No significant differences were observed in the number of pseudothecia between W1 and W2 (*P* > 0.05). a and a_1_ Cross mating of *ChΔmat0* {*UbMAT*}*-2*× *ChΔmat0* {*UbMAT*}*-3*. b and b_1_ Self mating of *ChΔmat0* {*UbMAT*}-*4*. A few, tiny pigmented pseudothecia were discovered in a and a_1_ or b and b_1_. c and c_1_ Cross mating of *C. heterostrophus* C5× *ChΔmat0*{*UbMAT*}-*3*. d and d_1_ cross mating of *C. heterostrophus* C4× *ChΔmat0* {*UbMAT*}-*2*. A medium number of slightly large pigmented pseudothecia were discovered in c and c_1_ or d and d_1_. e and e_1_ Cross mating of *C. heterostrophus* C5× *C. heterostrophus* C4 (**W1**). f and f_1_ Self mating of *C. heterostrophus* (2847) (W2). The maximum number of the largest pigmented pseudothecia were discovered in e and e1 or f and f_1_.

To test whether a mating phenotype was conferred by *U. botrytis MAT* transgenes expressed in the heterothallic *C. heterostrophus*, we conducted three cross matings *ChΔmat0* {*UbMAT*}*-2* × *ChΔmat0*{*UbMAT*}*-3*, *C. heterostrophus* C5× *ChΔmat0*{*UbMAT*}-*3* and *C. heterostrophus* C4× *ChΔmat0*{*UbMAT*}-*2* (Table S2) and one test of self-fertilization *ChΔmat0*{*UbMAT*}-4 (Table S2). As a result, numerous and tiny pigmented pseudothecia were produced by a cross mating *ChΔmat0*{*UbMAT*}*-2*× *ChΔmat0*{*UbMAT*}*-*3 that were very similar to those of a self mating of *ChΔmat0*{*UbMAT*}-*4* (*P* > 0.05) on the surface of corn leaf substrates (Fig. 5A,B: a,b, Table S2). Note that *ChΔmat0*{*UbMAT*}*-*2 and *ChΔmat*0{*UbMAT*}*-3* contain *U. botrytis MAT*1*-1-1* or *MAT1-2-1*, while *ChΔmat0*{*UbMAT*}-4 contain *U. botrytis MAT1-1-1* and *MAT1-2-1* (Table S2). In addition, two other cross matings, *C. heterostrophus* C5× *ChΔmat0*{*UbMAT*}-3 and *C. heterostrophus* C4× *ChΔmat0*{*UbMAT*}-2, produced almost the same numerous and slightly larger pigmented pseudothecia on the surface of corn leaf substrates (*P* > 0.05) (Fig. 5A,B: c,d, Table S2). For the two other cross matings, half were crossed to a transgenic strain carrying *C. heterostrophus MAT1-1-1* or *MAT1-2-1*, and half were crossed to a transgenic strain carrying *U. botrytis MAT1-1-1* or *MAT1-2-1*. As shown in Fig. 5A,B (a,b,c,d,e,f), the number and the sizes of the pigmented pseudothecia were gradually increased or enlarged on the surface of corn leaf substrates, respectively. Interestingly, no asci were noted when all the pseudothecia from self or cross mating strains were examined (Table S2, Fig. 5A: a_1_,b_1_,c_1_,d_1_) compared with those of WT1 (Fig. 4A: g,g_1_, Fig. 4B,C: g. Table S2) and WT2 (Fig. 4A: h and h_1_, Fig. 4B,C: h, Table S2). These data demonstrate that the heterologous *U. botrytis MAT* genes are not only strongly expressed in the *C. heterostrophus* transgenic strains but also have the ability to induce a sexual mode of reproduction in the genetic background of the heterothallic *C. heterostrophus* strains.

## Discussion

Fungi are a group historically considered to present a high proportion of asexual species; a fifth of the species were once thought to exclusively reproduce asexually^41^. For example, most species in the filamentous ascomycetes genera *Alternaria*, *Stemphylium*, and *Ulocladium* are only known to reproduce asexually^29, 30^. Possible reasons for the absence of sex are that the suitable factors and conditions needed to induce sex have not been determined and further research needs to be conducted to identify suitable environmental conditions for sex, or that these fungal genomes lack the equipment to engage in sex^42, 43^. However, *MAT* genes have also been cloned and characterized from putatively asexual fungi and have been shown to be functional when expressed in closely related sexual species^19, 24^, even when they have not been demonstrated to function in the asexual progenitor. In asexual fungi, the functions of mating-type genes have proven particularly useful in molecular phylogenetic studies^24, 44, 45^. Our previous study demonstrated that the *MAT* genes are suitable for phylogenetic analysis for the four closely allied genera *Ulocladium*, *Alternaria*, *Cochliobolus*, and *Stemphylium* ^30^ and support a similar functional role in all four asexual genera. In this study, our experiments have demonstrated that *U. botrytis MAT1-1-1* and *MAT1-2-1* could influence colony growth and conidia size and number when deleted in *U. botrytis* or when expressed in *C. heterostrophus* (Figs 1 and 3) and that *C. heterostrophus MAT1-1-1* and *MAT1-2-1* could also exert similar effects when expressed in *U. botrytis* (Fig. 2). Thus, the mating-type genes in these two closely related fungi are functional and influence both sexual and asexual characteristics. The presence of mating-type genes in both taxa with and without a known sexual stage allow these genetic characters to be integrated across both anamorphs and teleomorphs and are particularly useful for consolidating the taxonomy of these two groups^46^.

Several putatively asexual species have been previously reported to contain functional, constitutively transcribed *MAT* genes^19, 36, 39^. Among these species are plant pathogens such as *A. alternata*, *S. herbarum*, *S. triglochinicola* and *S. eturmiunm*, as well as biotechnologically relevant anamorphic fungi, including *Aspergillus fumigatus*, and *Penicillium marneffei* ^24, 47, 48^. Analyses of the *MAT* gene sequences of these asexual fungi revealed the presence of transcriptionally active *MAT* genes which are normally associated with sexual reproduction^47, 48^. These reports indicate that the absence of detectable sexual reproduction in the asexual filamentous ascomycetes is not due to the lack of mating-type genes nor is it due to the occurrence of disruptive mutations within *MAT* genes or other sex-related genes. Thus, sexual reproduction in the filamentous ascomycetes is universally genetically controlled by a sex-specific region referred to as the mating-type locus^1, 2^. Our previous study demonstrated that all *Ulocladium* species usually carry both *MAT1-1-1* and *MAT1-2-1* in a single genome, which provides further evidence supporting that all *Ulocladium* species may have the potential to reproduce sexually during the life cycle^30^. However, no sexual state has yet been identified for *Ulocladium*, which is therefore thought to be a strictly asexual filamentous ascomycete genus. It is possible that the *MAT* genes within *Ulocladium* species can not effectively regulate sexual reproduction. The *U. botrytis MAT1-1-1* and *MAT1-2-1* sequences are homologous to *MAT-1-1-1* and *MAT1-2-1* of the related heterothallic species *C. heterostrophus*. The coding sequences of the α-box domain of both *MAT1-1-1* genes (Fig. S1) and HMG-box domain of both *MAT1-2-1* genes (Fig. S2), apart from their 47 or 43 nonhomologous sequences, are 71.43% or 72.44% identical, respectively. The *U. botrytis MAT1-1-1* and *MAT1-2-1* sequences are thus lowly similar to those of *C. heterostrophus MAT-1-1* and *MAT1-2-1*, respectively. When either of the *C. heterostrophus MAT1-1-1* or *MAT1-2-1* genes were transformed into *U. botrytis*, the recipient could neither self nor cross with other *U. botrytis* strains, in contrast to wild type *C. heterostrophus* strains and transgenic *C. heterostrophus* strains which can do both. Notably, all the mating patterns of the transgenic *U. botrytis* strains containing *U. botrytis* genes did not induce sexual reproduction (Table S2, Fig. 4A). On the other hand, introduction of the *U. botrytis MAT1-1-1* and *MAT1-2-1* into the *C. heterostrophus* C4, C5, and C4-41.7 strains induced either by self-mating or cross-mating a varying degree sexual reproduction (Table S2, Fig. 5A), suggesting the *U. botrytis MAT* genes have not lost the ability for initiating sexual reproduction. Thus, the lack of sexual reproduction in *U. botrytis* is not due to either absence or mutation of *MAT* genes, as was observed for *A. alternata* and *B. sacchari* ^24^, nor is it due to the low similarity of the *MAT1-1-1* and *MAT1-2-1* sequences between *U. botrytis* and *C. heterostrophus* (Figs S1 and S2). We hypothesize that there are multiple possible reasons that *U. botrytis MAT* genes are not triggering sexual reproduction in the laboratory conditions tested. First, *MAT* genes encode transcriptional regulators that normally control the expression of many genes required for sexual reproduction, including the mating pheromones and their G-protein–coupled receptors^49^, and these *MAT*-regulated genes may have evolved to not control sexual reproduction in *U. botrytis*. Alternatively, the genetic background of *U. botrytis* may restrict the roles of *MAT* genes in sexual reproduction to environmental conditions not tested here. However, another explanation is that *U. botrytis* may have a cryptic sexual cycle similar to the human pathogen *Coccidioides immitis* ^50^, but sexual reproduction may be a rare event that is hard to detect as it was for the presumed asexual barley pathogen *Septoria passerinii* ^51^ and thus remains to be described.

Mating-type genes have been characterized in a number of heterothallic and homothallic filamentous ascomycetes, where they function as master regulators of sexual reproduction^52^. *MAT* genes govern both the ability of a strain to undergo sexual reproduction but are also critical in the evolution of heterothallic and homothallic modes of mating by exchange or rearrangement of *MAT* genes^52–55^. In this study, we addressed the function of *MAT* genes of *U. botrytis* by expressing heterothallic *C. heterostrophus MAT1–1–1* or *MAT1-2-1* genes in single or a double *MAT*-deleted *U. botrytis* strains and evaluating if the *C. heterostrophus MAT* genes could promote sexual reproduction in *U. botrytis*. Unexpectedly, our results demonstrate that both *ChMAT1-1-1* and *ChMAT1-2-1* could not trigger sexual reproduction in all transgenic *U. botrytis* strains despite the multiple tests of different mating specificity (Fig. 4A,B: a,b,c,d,e, Table S2), as observed in the wildtype *U. botrytis* strain (Fig. 4A,B: f, Table S2). However, the *MAT* genes of both *U. botrytis* and *C. heterostrophus* were shown to be able to influence asexual characteristics in both species. These observations are consistent with studies showing that expression of genes during asexual growth is also dependent on *MAT*, such as in isogenic *Neurospora crassa* and *Aspergillus oryzae* strains^4, 14^. *MAT* gene regulation of diverse functions has been observed in asexual fungi such as *Fusarium graminearum* ^56^, *Penicillium chrysogenum* ^57^ and in sexual fungi *Podospora anserina* ^58^, *Sordaria macrospora* ^59^ and *Neurospora crassa* ^18^, including metabolism, cell wall organization, cellular response to stimuli, cell adhesion, fertilization, information pathways, transport, and developmental processes. A broader understanding that *MAT* genes pleiotropically control both asexual and sexual reproduction is provided by these studies and our study on *U. botrytis*. For these reasons, the function of *MAT* genes in fungi with no known sexual cycle needs to be carefully scrutinized before concluding that they promote outcrossing and meiotic reproduction.

In all *C. heterostrophus* transgenic strains, the heterothallic transgenic *ChΔmat0*{*UbMAT*}-4 strain was changed to homothallic when *U. botrytis MAT1-1-1* and *MAT1-2-1* were co-introduced into the C4-41.7 (MAT_0_) strain, but all other *C. heterostrophus* transgenic strains still mated in a heterothallic manner, including crosses between *ChΔmat0* strains carrying complementary *U. botrytis MAT* genes (Table S2). Thus, all *C. heterostrophus* transgenic strains were able to cross in a heterothallic manner or self in a homothallic manner using the *U. botrytis* genes, although the phenotypes were different from those of the genetic background of *C. heterostrophus*. Specifically, all these self and cross phenotypes were able to produce fewer and smaller pseudothecia (Fig. 5A: a,b,c,d, Table S2) but were not able to produce asci compared to those of wild type *C. heterostrophus* crosses (Fig. 5A: e,f, Table S2). These observations suggest that partial characteristics of sexual reproduction in these *C. heterostrophus* transgenic strains are attributable to the introduction of *U. botrytis MAT* genes into the genetic background of the heterothallic *C. heterostrophus*. Thus, these results suggest that the genetic backgrounds of the *C. heterostrophus* and *U. botrytis* strains may play significant roles in determining the potential effect of *MAT* genes on sexual reproduction in heterothallic and homothallic strains. In summary, this study reveals that *U. botrytis MAT1-1-1* and *MAT1-2-1* may have not lost the ability for sexual reproduction in this species which has only been observed reproduce asexually and that the *MAT* genes play a major role in controlling asexual characteristics.

## Methods

### Strains, culture conditions, and crosses

The *U. botrytis* strain^29^ (CBS 198.67) (*MAT1-1-1*: KF533878, *MAT1-2-1*: KF533888)^30^ was grown on potato carrot agar (PCA) under standard conditions^33^. Some test strains, including *C. heterostrophus* strains C5 (ATCC48332) only containing *MAT1-1-1* (X68399), C4 (ATCC48331) only containing *MAT1-2-1* (X68398), *C. heterostrophus* strain 2847 carrying *MAT1-2-1/1-1-1*, and a double mat-deleted C4-41.7 (MAT_0_) strain, were obtained from O. C. Yoder and B. G. Turgeon of Cornell University (Ithaca, NY, U.S.A). Note that the C4-41.7 strain is derived from C4 that lacks the whole mating-type locus^60^. These test strains were cultured on complete medium with xylose (CMX)^11^ and incubated under 16 h light/8 h dark at approximately 22 °C for 12 days. In this study, selfing or crossing of *U. botrytis*, *C. heterostrophus* and all transgenic strains were performed using procedures previously described for *C. heterostrophus* ^32, 61^.

### Amino acid alignment and phylogenetic analysis

Assembled *U. botrytis MAT1-1-1* and *MAT1-2-1* sequences were aligned with *MAT1-1-1* and *MAT1-2-1* sequences from *C. heterostrophus* (X68399, X68398, respectively), *A. alternata* (AB009451, AB009452, respectively) and *S. eturmiunum* (EGS29-099, EGS29-099, respectively). Assembled sequences were analyzed for putative open reading frames and introns using Genetyx Mac v.11.2 software (Genetyx, Shibuya, Tokyo, Japan). Putative introns were spliced from the open reading frames, conceptually translated using Jellyfish software (Lab Velocity, San Francisco, CA), and aligned in ClustalX BLAST^62^ searches for similar nucleotide and protein sequences were carried out against the National Center for Biotechnology Information (NCBI) databases.

### Deletion of *MAT1-1-1* and *MAT1-2-1* of homothallic *U. botrytis*

Fungal transformation and molecular characterization of gene knockout mutants were conducted according to Leng *et al*.^63^. The split-marker system^64^ was used for gene deletion, and *hygB* ^R^ or G418 transformants were purified by successive transfer of young hyphal tips of *U. botrytis* to selective medium and screened for self-sterility. The *MAT1-1-1* and *MAT1-2-1* genes in the asexual *U. botrytis* were identified in a previous study^30^. *U. botrytis MAT1-1-1* or *MAT1-2-1* was deleted using the split-marker method, with the exception that the entire selectable marker cassette was amplified from plasmid pUCATPH^65^, then fused to the 5′ and 3′ flanking fragments of the *MAT1-1-1* or *MAT1-2-1*. Transformation was conducted following a described protocol^66^. Single mutant *ΔmatUbMAT-1* or *ΔmatUbMAT-2* was individually constructed as shown in Table S1. The double mutant *DmUbMAT-1* (*ΔmatUbMAT1-1-1/1-2-1*) was constructed by deletion of *UbMAT1-2-1* from the single mutant *ΔmatUbMAT-1* (Table S1). For the deletion, the 5′ and 3′ flanking fragments of *MAT1-2-1* were fused to the *NPTII* selectable marker cassette from pII99^67^ by overlapping PCR, and the fused fragment was used for transformation of the *ΔmatUbMAT-1* strain (Table S1). Transformants were subjected to RT-PCR, Southern blot and Northern blot analysis to confirm deletion of *MAT1-1-1, MAT1-2-1*, and *MAT1-1-1/1-2-1* which were performed as described below. *ΔmatUbMAT-1* strain was chosen as the recipient for heterologous expression of *C. heterostrophus MAT1-1-1 ΔmatUbMAT-2* strain was chosen as the recipient for heterologous expression of *C. heterostrophus MAT1-2-1. DmUbMAT-1* was chosen as the recipient for heterologous expression of *C. heterostrophus MAT1-1-1*/*1-2-1*.

### Transformation of *C. heterostrophus* and *U. botrytis*

Plasmid pBG, carrying *bar*-encoding resistance to *hygB* ^R^ ^68^, was obtained from Tsutomu Arie^19^. For transformation procedures, *C. heterostrophus* C4-41.7 (MAT_0_), *DmUbMAT-1*, *ΔmatUbMAT-*1, and *ΔmatUbMAT-*2 strains were cultivated as described above. The preparation of *C. heterostrophus* C4-41.7, *ΔmatUbMAT-*1, *ΔmatUbMAT-*2, and *DmUbMAT-1* protoplasts was performed as described previously^14, 66^. *Bar* ^R^ transformants were selected on a selective regeneration medium. The segregation of antibiotic-resistant phenotypes in the sexual crosses was then scored on PCA or CMX medium.

### Crossing: determination of mating phenotypes of *U. botrytis* transgenic strains carrying *C. heterostrophus MAT* genes

*U. botrytis* transgenic strains carrying opposite *C. heterostrophus MAT* genes were crossed and selfed as indicated in Table S2. The unsuccessful crosses were as follows: *DmUbMAT-1*{*ChMAT*}*-1-1*× *DmUbMAT-1*{*ChMAT*}*-2-1*, and *ΔmatUbMAT-1*× *ΔmatUbMAT-2*. The successful self matings were as follows: *U. botrytis* strain (Wild type), *DmUbMAT-1*{*ChMAT*}*-3-1, ΔmatUbMAT-1*{*ChMAT*}*-1*, and *ΔmatUbMAT-2*{*ChMAT*}*-1*. The negative controls were as follows: a self-mating *U. botrytis* strain and a cross mating *ΔmatUbMAT-1*× *ΔmatUbMAT-2*. The positive controls were as follows: a self-mating *C. heterostrophus* (2847) and a cross mating *C. heterostrophus* C5 × *C. heterostrophus* C4. All cross and self-mating strains were cultured on the corn leaf substrate as described above. Fertility from self or cross mating was determined by checking the number of pseudothecia per square centimeter of area on the corn leaf substrates, the number of asci in individual pigmented pseudothecia, and the number of ascospores in individual asci. For the initial screening, at least 10 pseudothecia were opened and the number of asci per pseudothecium were recorded. Each experiment was repeated at least three times.

### Crossing: determination of mating phenotypes of *C. heterostrophus* transgenic strains carrying *U. botrytis MAT* genes

The transgenic strains *ChΔmat0*{*UbMAT*}-2, *ChΔmat0*{*UbMAT*}-3 and *ChΔmat0* {*UbMAT*}-4 carrying *U. botrytis MAT1-1-1* or *MAT1-2-1* were mated in pairs as indicated in Table S1. One cross was performed with a heterothallic *MAT* gene pattern: *ChΔmat0* {*UbMAT*}-2 was crossed to *ChΔmat0*{*UbMAT*}-3 on the surface of corn leaf substrates. Control cross patterns: *C. heterostrophus* C5 was crossed to *C. heterostrophus* C4; *ChΔmat0*{*UbMAT*}-4 or *C. heterostrophus* (2847) was individual selfed. Fertility from self or cross mating was determined by checking the number of pigmented pseudothecia per square centimeter on the surface of corn leaf substrates, the number of asci in individual pseudothecia, and the number of ascospores in individual asci. For the initial screening, at least 10 pseudothecia were opened and the number of asci in each pseudothecium were recorded. Each experiment was repeated at least three times.

### Nucleic acid manipulation

*U. botrytis* strain cultivation and DNA extraction were conducted as previously described^30^. *C. heterostrophus* strain growth and genomic DNA purification followed the procedures described by Turgeon *et al*.^11^. Total RNA was extracted using the TRrizol reagent (Invitrogen, USA) according to the manufacturer’s protocol. PCR amplifications were performed in a total volume of 20 μl containing 0.4 μM of each dNTP, 5 μM of each primer, 1 unit of easy *Taq* or 2 units easy *Pfu* DNA polymerase (Trans, China), 2.0 μl of 10 reaction buffer, and 10 to 20 ng of genomic DNA. Southern blotting and Northern blotting were adjusted slightly according to previous descriptions^69^. For Southern blot analysis of *MAT* genes in the transgenic strains of *U. botrytis* deletion lines and *C. heterostrophus* C5, C4 and C4-41.7 (*MAT*_*0*_)*, MAT*-specific probes were prepared by PCR amplification (Table S3) of *MAT1-1-1* and *MAT1-2-1* from *U. botrytis* strain (CBS 198.67), *C. heterostrophus* strains C5 and C4, respectively, using primers UMAT1-1F and UMAT1-1R to amplify *MAT1-1-1* from *U. botrytis*, and UMAT1-2F and UMAT1-2R to amplify *MAT1-2-1* from *U. botrytis*; using primers CMAT1-1F and CMAT1-1R to amplify *MAT1-1-1* from C5, and CMAT1-2F and CMAT1-2R to amplify *MAT1-2-1* from C4. For Southern blot analysis of *MAT* deletion lines in *U. botrytis*, both *hygB* and G418 probes were used detect transgene insertion. PCR amplicons were column purified and approximately 1 μg of DNA was random prime labeled with digoxigenin-11-dUTP using the DIG DNA Labeling and Detection Kit (Roche Diagnostics, Indianapolis, IN) according to the manufacturer’s instructions. Hybridization, washing, and chemiluminescent detection with CSPD were carried out with the same kit. Hybridization was detected by exposing the membranes to Kodak X-OMAT film (Kodak, Rochester, NY) for 15–30 min and developed under standard conditions. The Northern blot was also adjusted slightly according to previous descriptions^69^.

The expression of *U. botrytis MAT1-1-1* and *MAT1-2-1* loci in *C. heterostrophus MAT* deletion lines and *C. heterostrophus* C5, C4 and C4-41.7 strains was analyzed for RNA expression using qRT-PCR. RT-PCR was performed with the PrimeScript strand cDNA Synthesis Kit (Takara, Japan) following the supplier’s instructions. Transcript levels were quantitated using either the threshold cycle (ΔΔCT) method or a relative standard curve. SYBR green sequence detection was performed using the StepOne real-time PCR system (Applied Biosystems)^70^. To monitor the expression of *U. botrytis MAT1-1-1* or *MAT1-2-1* in reference transgenic *C. heterostrophus* strains C5, C4 and C4-41.7, we used the primers listed in Table S3. The *C. heterostrophus* actin gene (AY748990) was used as the endogenous control to normalize the expression of *MAT1-1-1* or *MAT1-2-1* in all transgenic lines of *C. heterostrophus*. To monitor the expression of the *C. heterostrophus MAT1-1-1* or *MAT1-2-1* in reference transgenic *U. botrytis* strains, we used the primers listed in Table S3. The actin gene was used as the endogenous control to normalize the expression of *MAT1-1-1* or *MAT1-2-1* genes in all transgenic lines of *U. botrytis. Actin*-F and *Actin*-R primers were used to amplify the actin gene in all tested strains (Table S3). Validation experiments of target genes and control genes for the comparative ΔΔCT method were performed according to the instructions of Applied Biosystems^70^. For a valid ΔΔCT method calculation, the efficiency of the target amplification and the efficiency of reference amplification must be approximately equal. Relative quantitation is expressed as a difference in target gene expression with respect to an endogenous control in different samples. Each cDNA sample was assayed in triplicate, and RNAs were obtained from three separate biological samples.

### Light Microscopy

For microscopic studies, all transformants, *C. heterostrophus* or *U. botrytis* wild-type and *MAT*-deleted strains were cultivated using standard conditions^11, 32^. Microscopy was performed using an Olympus BX-53 microscope (Tokyo, Japan). The preparations of fruiting bodies and asexual spores of *C. heterostrophus* or *U. botrytis* were conducted following the procedures described by Wang *et al*.^32^ and Turgeon *et al*.^11^. The pseudothecia and asci produced by the different transformants from the cross or self matings were stained with cotton blue. Photographs were subsequently processed using the Autolevel and Autocontrast features of Adobe Photoshop 9.0. Each experiment was repeated at least three times.

## Electronic supplementary material

Supplementary Information

## References

[1] Coppin E, Debuchy R, Arnaise S, Picard M (1997). Mating types and sexual development in filamentous ascomycetes. Microbiol Mol Biol Rev..

[2] Turgeon BG (1998). Application of mating type gene technology to problems in fungal biology. Ann Rev Phytopathol..

[3] Turgeon BG, Yoder OC (2000). Proposed nomenclature for mating type genes of filamentous ascomycetes. Fungal Genet Biol..

[4] Kim H, Borkovich KA (2004). A pheromone receptor gene, pre-1, is essential for mating type-specific directional growth and fusion of trichogynes and female fertility in *Neurospora crassa*. Mol Microbiol..

[5] Mayrhofer S, Weber JM, Pöggeler S (2006). Pheromones and pheromone receptors are required for proper sexual development in the homothallic ascomycete *Sordaria macrospora*. Genetics.

[6] Pöggeler S (2000). Two pheromone precursor genes are transcriptionally expressed in the homothallic ascomycete *Sordaria macrospora*. Curr Genet..

[7] Yun SH, Arie T, Kaneko I, Yoder OC, Turgeon BG (2000). Molecular organization of mating type loci in heterothallic, homothallic, and asexual *Gibberella*/*Fusarium* species. Fungal Genet Biol..

[8] Kronstad JW, Staben C (1997). Mating type in filamentous fungi. Ann Rev Genet..

[9] Pyrzak W, Miller KY, Miller BL (2008). Mating type protein Mat1-2 from asexual *Aspergillus fumigatus* drives sexual reproduction in fertile *Aspergillus nidulans*. Eukaryotic Cell.

[10] Klix V (2010). Functional characterization of MAT1-1-specific mating-type genes in the homothallic ascomycete *Sordaria macrospora* provides new insights into essential and nonessential sexual regulators. Eukaryotic Cell.

[11] Turgeon BG (1993). Cloning and analysis of the mating type genes from *Cochliobolus heterostrophus*. Mol Gen Genet..

[12] Paoletti M (2007). Mating type and the genetic basis of self-fertility in the model fungus *Aspergillus nidulans*. Curr Biol..

[13] Pöggeler S, Hoff B, Kück U (2008). Asexual cephalosporin C producer *Acremonium chrysogenum* carries a functional mating type locus. Appl Environ Microbiol..

[14] Wada, R. *et al*. Presence and functionality of mating type genes in the supposedly asexual filamentous fungus *Aspergillus oryzae*. *Appl Environ Microbiol*. 2819–2829 (2012). 10.1128/AEM.07034-11 PMC3318824 22327593

[15] Yi S (2011). Alternative mating type configurations (a/a versus a/a or a/a) of *Candida albicans* result in alternative biofilms regulated by different pathways. PLoS Biol..

[16] Hicks J, Strahern JN, Klar AJS (1979). Transposable mating type genes in *Saccharomyces cerevisiae*. Nature.

[17] Reynolds, D. R. & Taylor, J. W. The fungal holomorph: mitotic, meiotic and pleomorphic speciation in fungal systematics. *Wallingford, UK: CAB Int* (1993).

[18] Wang Z, Kin K, López-Giráldez F, Johannesson H, Townsend JP (2012). Sex-specific gene expression during asexual development of *Neurospora crassa*. Fungal Genet Biol..

[19] Arie T (2000). Mating-type genes from asexual phytopathogenic ascomycetes *Fusarium oxysporum* and *Alternaria alternata*. Mol Plant Microbe Interact..

[20] Dyer, P. S. Sexual reproduction and significance of *MAT* in the aspergilli, p. 123–142. *In* Heitman J., Kronstad, J. W., Taylor, J. W., Casselton, L. A. (ed) Sex in fungi: molecular determination and evolutionary implications. ASM Press Washington DC **(**2007).

[21] Galagan JE (2005). Sequencing of *Aspergillus nidulans* and comparative analysis with *A. fumigatus* and *A. oryzae*. Nature.

[22] Kerónyi Z, Moretti A, Waalwijk C, Oláh B, Hornok L (2004). Mating type sequences in asexually reproducing *Fusarium* species. Appl Environ Microbiol..

[23] Pöggeler S (2002). Genomic evidence for mating abilities in the asexual pathogen *Aspergillus fumigatus*. Curr Genet..

[24] Sharon A (1996). An asexual fungus has the potential for sexual development. Mol Gen Genet..

[25] Woo PC (2006). Genomic and experimental evidence for a potential sexual cycle in the pathogenic thermal dimorphic fungus *Penicillium marneffei*. FEBS Lett..

[26] Berbee ML, Payne BP, Zhang G, Roberts RG, Turgeon BG (2003). Shared ITS DNA substitutions in isolates of opposite mating type reveal a recombining history for three presumed asexual species in the filamentous ascomycete genus. Alternaria. Mycol Res..

[27] Paoletti M (2005). Evidence for sexuality in the opportunistic fungal pathogen *Aspergillus fumigatus*. Curr Biol..

[28] Bolton MD (2012). Evaluation of the potential for sexual reproduction in field populations of *Cercospora beticola* from USA. Fungal Biol..

[29] Simmons EG (1967). Typification of *Alternaria*, *Stemphylium*, and *Ulocladium*. Mycologia.

[30] Geng Y (2014). Characterization and phylogenetic analysis of the mating-type loci in the asexual ascomycete genus *Ulocladium*. Mycologia.

[31] Zhang XG, Zhang TY (2002). Studies on the genus *Ulocladium* Preuss from China. Mycosystema.

[32] Wang Y, Pei YF, O’Neill NR, Zhang XG (2010). *Ulocladium cantlous* sp. nov. isolated from northwestern China: its morphology and molecular phylogenetic position. Mycologia.

[33] Wang Y, Bruno LC, Zhang XG (2008). Two new species of *Ulocladium* from Southwest China. Mycologia.

[34] Lucas MT, Webster J (1964). Conidia of *Pleospora scirpicola* and *P. valesiaca*. Trans Br Mycol Soc..

[35] Simmons EG (2001). Perfect States of *Stemphylium* IV. Harvard Pap Bot..

[36] Webster J (1969). The Pleospora state of *Stemphylium triglochinicola*. Trans Br Mycol Soc..

[37] Simmons EG (1985). Perfect states of *Stemphylium* II. Sydowia Ann Mycol Ser II.

[38] Simmons EG (1969). Perfect states of *Stemphylium*. Mycologia.

[39] Inderbitzin P, Harkness J, Turgeon BG, Berbee ML (2005). Lateral transfer of mating system in *Stemphylium*. Proc Natl Acad Sci USA..

[40] Bushley KE (2013). Isolation of the *MAT1-1* mating type idiomorph and evidence for selfing in the Chinese medicinal fungus *Ophiocordyceps sinensis*. Fungal Biol..

[41] Taylor JW, Jacobson D, Fisher M (1999). The evolution of asexual Fungi: reproduction, speciation and classification. Ann Review Phytopathol..

[42] Sugui JA (2011). Identification and characterization of an *Aspergillus fumigatus* “supermater” pair. mBio.

[43] Selker E, Cambareri EB, Jensen BC, Haack K (1987). Rearrangement of duplicated DNA in specialized cells of *Neurospora*. Cell.

[44] Pöggeler S (1999). Phylogenetic relationships between mating-type sequences from homothallic and heterothallic ascomycetes. Curr Genet..

[45] Voigt K, Cozijnsen AJ, Kroymann J, Pöggeler S, Howlett BJ (2005). Phylogenetic relationships between members of the crucifer pathogenic *Leptosphaeria maculans* species complex as shown by mating type (MAT1-2), actin and beta-tubulin sequences. Mol Phylogenet Evol..

[46] Reynolds DR, Taylor JW (1992). Article 59: reinterpretation or revision?. Taxon.

[47] Pöggeler S (2002). Genomic evidence for mating abilities in the asexual pathogen *Aspergillus fumigatus*. Curr Genet..

[48] Woo PC (2006). Genomic and experimental evidence for a potential sexual cycle in the pathogenic thermal dimorphic fungus *Penicillium marneffei*. FEBS Lett..

[49] Debuchy, R., Berteaux-Lecellier, V. & Silar, P. Mating systems and sexual morphogenesis in Ascomycetes, pp. 501–535 in cellular and molecular biology of filamentous fungi, edited by Borkovich, K. A., Ebbole, D.J., Press, A.S.M., Washington, D.C. (2010).

[50] Burt A, Carter DA, Koenig GL, White TJ, Taylor JW (1996). Molecular markers reveal cryptic sex in the human pathogen *Coccidioides immitis*. Proc Natl Acad Sci USA.

[51] Ware SB (2007). Discovery of a functional *Mycosphaerella* teleomorph in the presumed asexual barley pathogen *Septoria passerinii*. Fungal Genet Biol..

[52] Lu SW, Yun SH, Lee T, Turgeon BG (2011). Altering sexual reproductive mode by interspecific exchange of MAT loci. Fungal Genet Biol..

[53] Poeggeler S, Risch S, Kueck U, Osiewacz HD (1997). Mating-type genes from the homothallic fungus *Sordaria macrospore* are functionally expressed in a heterothallic ascomycete. Genetics.

[54] Glass NL, Smith ML (1994). Structure and function of a mating-type gene from the homothallic species *Neurospora africana*. Mol Gen Genet..

[55] Yun SH, Turgeon BG (1999). Molecular comparison of mating-type loci and adjacent chromosomal regions from self-fertile and self-sterile *Cochliobolus* species. Plant Pathol J..

[56] Kim HK (2015). A Large-scale functional analysis of putative target genes of mating-type loci provides insight into the regulation of sexual development of the cereal pathogen *Fusarium graminearum*. PLoS Genet..

[57] Böhm J (2013). Sexual reproduction and mating-type–mediated strain development in the penicillin-producing fungus *Penicillium chrysogenum*. Proc Natl Acad Sci USA..

[58] Bidard F (2011). Genome-wide gene expression profiling of fertilization competent mycelium in opposite mating types in the heterothallic fungus *Podospora anserina*. PLoS One.

[59] Klix V (2010). Functional characterization of MAT1-1-specific mating-type genes in the homothallic ascomycete *Sordaria macrospora* provides new insights into essential and nonessential sexual regulators. Eukaryot Cell.

[60] Wirsel S, Turgeon BG, Yoder OC (1996). Detection of the *Cochliobolus heterostrophus* mating-type (MAT) locus promotes the function of MAT. Curr Genet..

[61] Leach J, Lang BR, Yoder OC (1982). Methods for selection of mutants and *in vitro* culture of *Cochliobolus heterostrophus*. J Gen Microbiol..

[62] Altschul SF, Gish W, Miller W, Myres EW, Lipman DJ (1990). Basic local alignment search tool. J Mol Biol..

[63] Leng Y (2011). Development of transformation and RNA-mediated gene silencing systems for functional genomics of *Cochliobolus sativus*. Mol Plant Pathol..

[64] Catlett N, Lee BN, Yoder O, Turgeon B (2003). Split-marker recombination for efficient targeted deletion of fungal genes. Fungal Genet Newsl..

[65] Lu SW (1994). Tagged mutations at the Tox1 locus of *Cochliobolus heterostrophus* using restriction enzyme-mediated integration. Proc Natl Acad Sci USA..

[66] Turgeon BG, Condon B, Liu J, Zhang N (2010). Protoplast transformation of filamentous fungi. Methods Mol Biol..

[67] Oide S (2006). NPS6, encoding a nonribosomal peptide synthetase involved in siderophore-mediated iron metabolism, is a conserved virulence determinant of plant pathogenic ascomycetes. Plant Cell.

[68] Straubinger B, Straubinger E, Wirsel S, Turgeon G, Yoder O (1992). Versatile fungal transformation vectors carrying the selectable *bar* gene of *Streptomyces hygroscopicus*. Fungal Genet Newsl..

[69] Sambrook, J., Fritsch, E. F. & Maniatis, T. Molecular Cloning: A Laboratory Manual. Cold Spring Harbor Laboratory Press, Cold Spring Harbor, NY (2001).

[70] Applied Biosystems Guide to performing relative quantitation of gene expression using real-time quantitative PCR. *Applied Biosystems*, *Foster City, CA* (2004).

