# Supplementary material for: Mating-type genes of the anamorphic fungus Ulocladium botrytis affect both asexual sporulation and sexual reproduction
Source: Sci Rep. 2017 Aug 11;7:7932. doi: 10.1038/s41598-017-08471-3 (PMC5554195; doi:10.1038/s41598-017-08471-3)
Supplement: Supplementary file 1 — Supplementary Information [file 41598_2017_8471_MOESM1_ESM.pdf]

Mating-type genes of the anamorphic fungus *Ulocladium botrytis* affect both asexual sporulation and sexual reproduction

Qun Wang[1+], Shi Wang[1+], Chen Lin Xiong[1], Timothy Y. James[2] & Xiu Guo Zhang[1]*

[1]Department of Plant Pathology, Shandong Agricultural University, 61, Daizong Street, Tai'an, Shandong, 271018, China, Shandong Provincial Key Laboratory for Biology of Vegetable Diseases and Insect Pests

[2]Department of Evolutionary Biology, University of Michigan, Ann Arbor, MI 48109 USA

[*]Corresponding author: E-mail zhxg@sdau.edu.cn

[+]These authors contributed equally to this work

Table S1. Strains used in this study.

Original strains[a]	Asci or ascospore progeny[b]	Genotype[c]
C. heterostrophus (X68399)	NO	*C. heterostrophus* strain carrying *MAT1-1-1*
C. heterostrophus (X68398)	NO	*C. heterostrophus* strain carrying *MAT1-2-1*
A. alternata (AB009451)	NO	*A. alternate* strain carrying *MAT1-1-1*
A. alternata (AB009452)	NO	*A. alternate* strain carrying *MAT1-2-1*
S. eturmiunum (EGS29-099)	Yes	*S. eturmiunum* train carrying *MAT1-1-1/1-2-1*
C. heterostrophus (2847)	Yes	*C. heterostrophus* strain carrying *MAT1-1-1/1-2-1*
C. heterostrophus C5 (ATCC48332, 2829)	NO	*ΔmatChMAT1-2-1*, *hygB*
C. heterostrophus C4 (ATCC48331, 2849)	NO	*ΔmatChMAT1-1-1*, G418
C. heterostrophus C4-41.7 (MAT_0)	NO	*Δmat ChMAT1-1-1/1-2-1*, *hygB*, G418
U. botrytis (CBS 198.67)	NO	*U. botrytis* strain carrying *MAT1-1-1/1-2-1*
ΔmatUbMAT-1	NO	*ΔmatUbMAT1-1-1*, G418
ΔmatUbMAT-2	NO	*ΔmatUbMAT1-2-1*, *hygB*
DmUbMAT-1	NO	*ΔmatUbMAT1-1-1/1-2-1*, *hygB*, G418
ΔmatUbMAT-1{*ChMAT*}-1	NO	*ΔmatUbMAT*-1 transformation *C. heterostrophus MAT1-1-1*, G418
ΔmatUbMAT-1{*ChMAT*}-2		
ΔmatUbMAT-1{*ChMAT*}-3		
ΔmatUbMAT-1{*ChMAT*}-4		
ΔmatUbMAT-2 {*ChMAT*}-1	NO	Strain of *ΔmatUbMAT-2* transformation *C. heterostrophus MAT1-2-1*, *hygB*
ΔmatUbMAT-2 {*ChMAT*}-2		
ΔmatUbMAT-2 {*ChMAT*}-3		
ΔmatUbMAT-2 {*ChMAT*}-4		
DmUbMAT-1 {*ChMAT*}-1-1	NO	Transgenic strain transformation *C. heterostrophus MAT1-1-1*, G418
DmUbMAT-1 {*ChMAT*}-1-2		
DmUbMAT-1 {*ChMAT*}-1-3		
DmUbMAT-1 {*ChMAT*}-1-4		
DmUbMAT-1 {*ChMAT*}-2-1	NO	Transgenic strain transformation *C. heterostrophus MAT1-2-1*, *hygB*
DmUbMAT-1 {*ChMAT*}-2-2		
DmUbMAT-1 {*ChMAT*}-2-3		
DmUbMAT-1 {*ChMAT*}-2-4		
DmUbMAT-1 {*ChMAT*}-3-1	NO	Transgenic strain transformation *C. heterostrophus MAT1-2-1* (*hygB*) and *MAT1-1-1* (G418)
DmUbMAT-1 {*ChMAT*}-3-2		
DmUbMAT-1 {*ChMAT*}-3-3		
DmUbMAT-1 {*ChMAT*}-3-4		
ChΔMAT0{*UbMAT*}-2	NO	Strain C4-41.7 (MAT_0) transformation *U. botrytis MAT1-1-1*, G418
ChΔMAT0{*UbMAT*}-3	NO	Strain C4-41.7 (MAT_0) transformation *U. botrytis MAT1-2-1*, *hygB*
ChΔMAT0{*UbMAT*}-4	Yes	Strain C4-41.7 (MAT_0) transformation *U. botrytis MAT1-2-1 /1-1-1*, *hygB,* G418

[a]All strains were generated in this study.

bIsolated from pseudothecia produced in self or cross mating of corresponding original transformants. These progeny were used as ‘‘purified’’ transformants and subjected to Southern blot or qRT-PCR analysis and/or mating specificity tests.

cUb=U. botrytis, Ch=C. heterostrophus, △mat=mat-deleted (MAT1-1-1, MAT1-2-1, MAT1-1-1/ MAT1-2-1), hygB =E. coli hygromycin B resistance gene.

Table S2. Crossing or selfing capability and fertility of transgenic strains carrying *MAT* genes.

Crossing or Selfing[a]	Heterothallic or Homothallic[b]	Cross or self mating patterns[c]	Fertility[d]	
			No. of pseudothecia	No. of asci
ChΔmat0 {*UbMAT*}*-2* ×*ChΔmat0* {*UbMAT*}*-3*	Heterothallic	*ChΔmat0*{*UbMAT*}*-2*: The C4-41.7 strain carrying *U. botrytis MAT1-1-1*<br>*ChΔmat0* {*UbMAT*}*-3*: The C4-41.7 strain carrying *U. botrytis MAT1-2-1*	11	0
ChΔmat0 {*UbMAT*}*-4*	Homothallic	The strain C4-41.7 carrying *U. botrytis MAT1-1-1/MAT1-2-1*	13	0
C. heterostrophus C5 × *ChΔmat0* {*UbMAT*}*-3*	Heterothallic	C5 strain carrying *C. heterostrophus MAT1-1-1*<br>*ChΔmat0* {*UbMAT*}*-3*: The C4-41.7 strain carrying *U. botrytis MAT1-2-1*	19	0
C. heterostrophus C4 × *ChΔmat0* {*UbMAT*}*-2*	Heterothallic	C4 strain carrying *C. heterostrophus MAT1-2-1*<br>*ChΔmat0* {*UbMAT*}*-2*: C4-41.7 strain carrying *U. botrytis MAT1-1-1*	20	0
C. heterostrophus (2847)	Homothallic	*C. heterostrophus* strain carrying *MAT1-2-1*/*MAT1-1-1*	>45	88.5
C. heterostrophus C5 × *C. heterostrophus*C4	Heterothallic	C5 strain carrying *C. heterostrophus MAT1-1-1*<br>C4 strain carrying *C. heterostrophus MAT1-2-1*	>40	80.5
DmUbMAT-1 {*ChMAT*}*-1-1* ×*DmUbMAT-1*{*ChMAT*}*-2-1*	Heterothallic	*DmUbMAT-1*{*ChMAT*}*-1-1*: *DmUbMAT-1* carrying *C. heterostrophus MAT1-1-1*<br>*DmUbMAT-1*{*ChMAT*}*-2-1*: *DmUbMAT-1* carrying *C. heterostrophus MAT1-2-1*	0	0
DmUbMAT-1 {*ChMAT*}*-3-1*	Homothallic	*DmUbMAT-1* carrying *C. heterostrophus MAT1-2-1*/ *MAT1-1-1*	0	0
ΔmatUbMAT-1 {*ChMAT*}*-1*	Homothallic	*ΔmatUbMAT-1* carrying *C. heterostrophus MAT1-1-1*	0	0
ΔmatUbMAT-2 {*ChMAT*}*-1*	Homothallic	*ΔmatUbMAT-2* carrying *C. heterostrophus MAT1-2-1*	0	0
U. botrytis strain (CBS 198.67)	Homothallic	The strain carrying *U. botrytis MAT1-2-1/1-1-1*	0	0
ΔmatUbMAT-1× *ΔmatUbMAT-2*	Heterothallic	*ΔmatUbMAT*-1 carrying *U. botrytis MAT1-2-1*<br>*ΔmatUbMAT-2* carrying *U. botrytis MAT1-1-1*	0	0

[a] Three replicates were set up for each self mating or crossing. *C. heterostrophus* (2847) and *C. heterostrophus* C5 (2829) ×*C. heterostrophus* C4 (2849) were used as negative controls. *U. botrytis* strain (CBS 198.67) and *ΔmatUbMAT-1*× *ΔmatUbMAT-2* were used as positive controls.

[b]The test for heterothallic or homothallic mating behavior.

[c] The strains of different mating patterns carrying the heterothallic *MAT* genes or the homothallic *MAT* genes.

[d] Fertility was estimated by counting the average number of pseudothecia per square centimeter of the corn leaf area (from three cross or self mating leaves) and average number of asci per pseudothecium (from 10 random pseudothecia). There were no differences in the number of ascospores produced in individual asci from each of the WT1 and WT2. No pseudothecia and asci were discovered from either of the *U. botrytis* strain and cross mating of *ΔmatUbMAT-1*×*ΔmatUbMAT-2*.

Table S3. Primers used in this study.

Primers	Sequence (5' to 3'): restriction sites underlined	Purpose
UMAT1-1-1F	ATGGACACTGCAGGTTTCGTTGC	Amplification of *MAT1-1-1* from
UMAT1-1-1R	TCAAGCGTTGGGAACATCGTCGAAT	*U. botrytis* (CBS 198.67)
UMAT1-2-1F	ATGAACGCAGAGATCTACCGCACT	Amplification of *MAT1-2-1* from
UMAT1-2-1R	CTAGTAGGTGTCCTGGAAGAAGGC	*U. botrytis* (CBS 198.67)
CMAT1-1-1F	ATGGCCCATGCAAGGGATCC	Amplification of *MAT1-1-1* from
CMAT1-1-1R	TCAAATGTGCGTGATGTCATCGA	*C. heterostrophus* C5 (ATCC48332)
CMAT1-2-1F	ATGGATTCTACAGTCTACTCTACTCCT	Amplification of *MAT1-2-1* from
CMAT1-2-1R	TTAGTAGGTGTCTTCGAACAAGGAC	*C. heterostrophus* C4 (ATCC48331)
MAT1-1-1F	ATGGACACTGCAGGTTTCGTTGC	PCR for *MAT-1-1-1* in *U. botrytis*
MAT1-1-1R	TCAAGCGTTGGGAACATCGTCGAAT	
MAT1-2-1F	ATGAACGCAGAGATCTACCGCACT	PCR for *MAT-1-2-1* in *U. botrytis*
MAT1-2-1R	CTAGTAGGTGTCCTGGAAGAAGGC	
CMAT1-1-1F	ATGGCCCATGCAAGGGATCC	PCR for *MAT-1-1-1* in *C. heterostrophus*
CMAT1-1-1R	TCAAATGTGCGTGATGTCATCGA	
CMAT1-2-1F	ATGGATTCTACAGTCTACTCTACTCCT	PCR for *MAT-1-2-1* in *C. heterostrophus*
CMAT1-2-1R	TTAGTAGGTGTCTTCGAACAAGGAC	
QMAT1-1-1F	GTGGGAAGCCGATCCAAA	qRT-PCR for *MAT1-1-1* in *U. botrytis*
QMAT1-1-1R	CTGGCGTAGGTATGTTGAGGTAAG	
QMAT1-2-1F	CCAGTCACGCAAGGCTAAG	qRT-PCR for *MAT1-2-1* in *U. botrytis*
QMAT1-2-1R	GTTGACGGGAAGTGCAGATT	
QCMAT1-1-1F	TGGACTCCTATGGGAAGCA	qRT-PCR for *MAT1-1-1* in *C. heterostrophus*
QCMAT1-1-1R	TAGTTTGAGGTGAGGCCAGA	
QCMAT1-2-1F	GTGGGCGAAGTTTGTGGGTT	qRT-PCR for *MAT1-2-1* in *C. heterostrophus*
QCMAT1-2-1R	GAAGGCGATGTCTGGGCTGA	
Actin-F	GGCAACATTGTCATGTCTGG	qRT-PCR fo*r Actin* gene in *U. botrytis*
Actin-R	GAGCGAAGCAAGAATGGAAC	
MAT1-1-1-Xho-F	CCG*CTCGAG*ATGGACACTGCAGGTTTCGT	Primers used for vector construction of *U. botrytis MAT1-1-1* heterologous expression in *C. heterostrophus*
MAT1-1-1-Hind-R	CCC*AAGCTT*AGCGTTGGGAACATCGTCGA	
MAT1-2-1-Xho-F	CCG*CTCGAG*ATGAACGCAGAGATCTACCG	Primers used for vector construction of *U. botrytis MAT1-2-1* heterologous expression in *C. heterostrophus*
MAT1-2-1-Hind-R	CCC*AAGCTT*GTAGGTGTCCTGGAAGAAGG	
MAT1-1-1-Xho-F	CCG*CTCGAG*ATGGCCCATGCAAGGGAT	Primers used for vector construction of *C. heterostrophus MAT1-1-1* heterologous expression in *U. botrytis*
MAT1-1-1-Hind-R	CCC*AAGCTT*AATGTGCGTGATGTCATCGA	
MAT1-2-1-Xho-F	CCG*CTCGAG*ATGGATTCTACAGTCTACTCTACTC	Primers used for vector construction of *C. heterostrophus MAT1-2-1* heterologous expression in *U. botrytis*
MAT1-2-1-Hind-R	CCC*AAGCTT*ATGGATTCTACAGTCTACTCTACTC	

All the restrict enzyme sites are in italic.

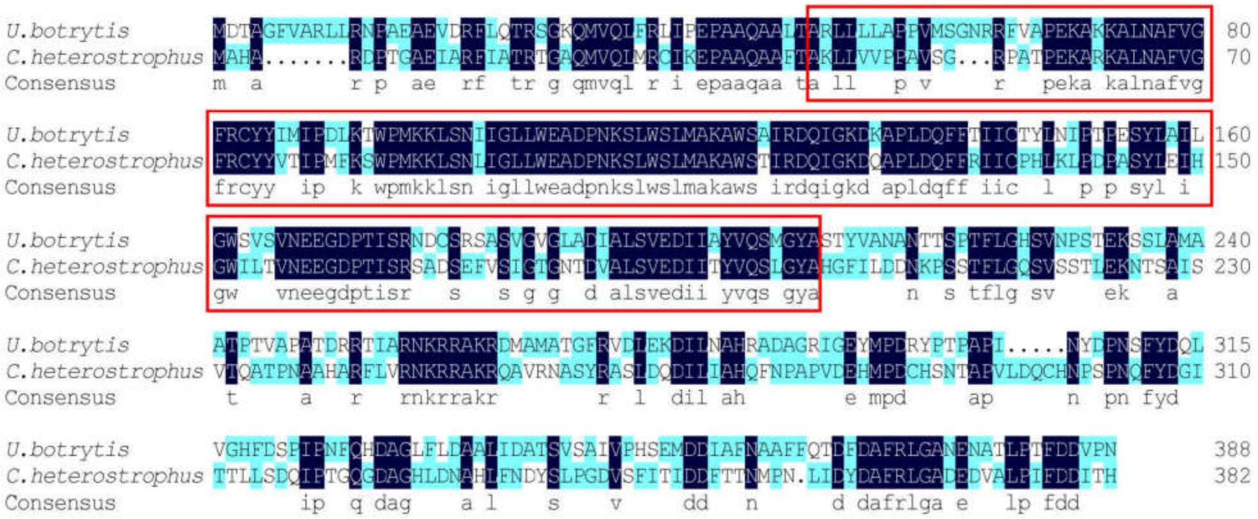

Figure S1. Amino acid sequence alignment of *MAT1-1-1* proteins from *U. botrytis*, and *C. heterostrophus*. The conserved α-box domain is boxed in red colour. Arrowheads indicate the locations of conserved introns.

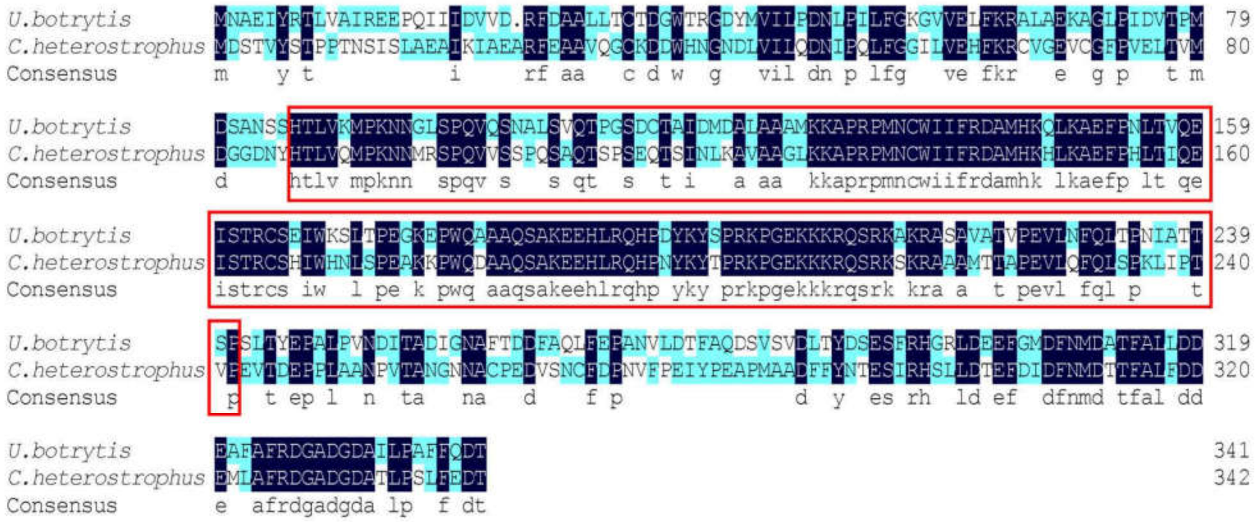

Figure S2. Amino acid sequence alignment of *MAT1-2-1* proteins from *U. botrytis*, and *C. heterostrophus*. The conserved HMG domain is boxed in red colour. Arrowheads indicate the locations of conserved introns.

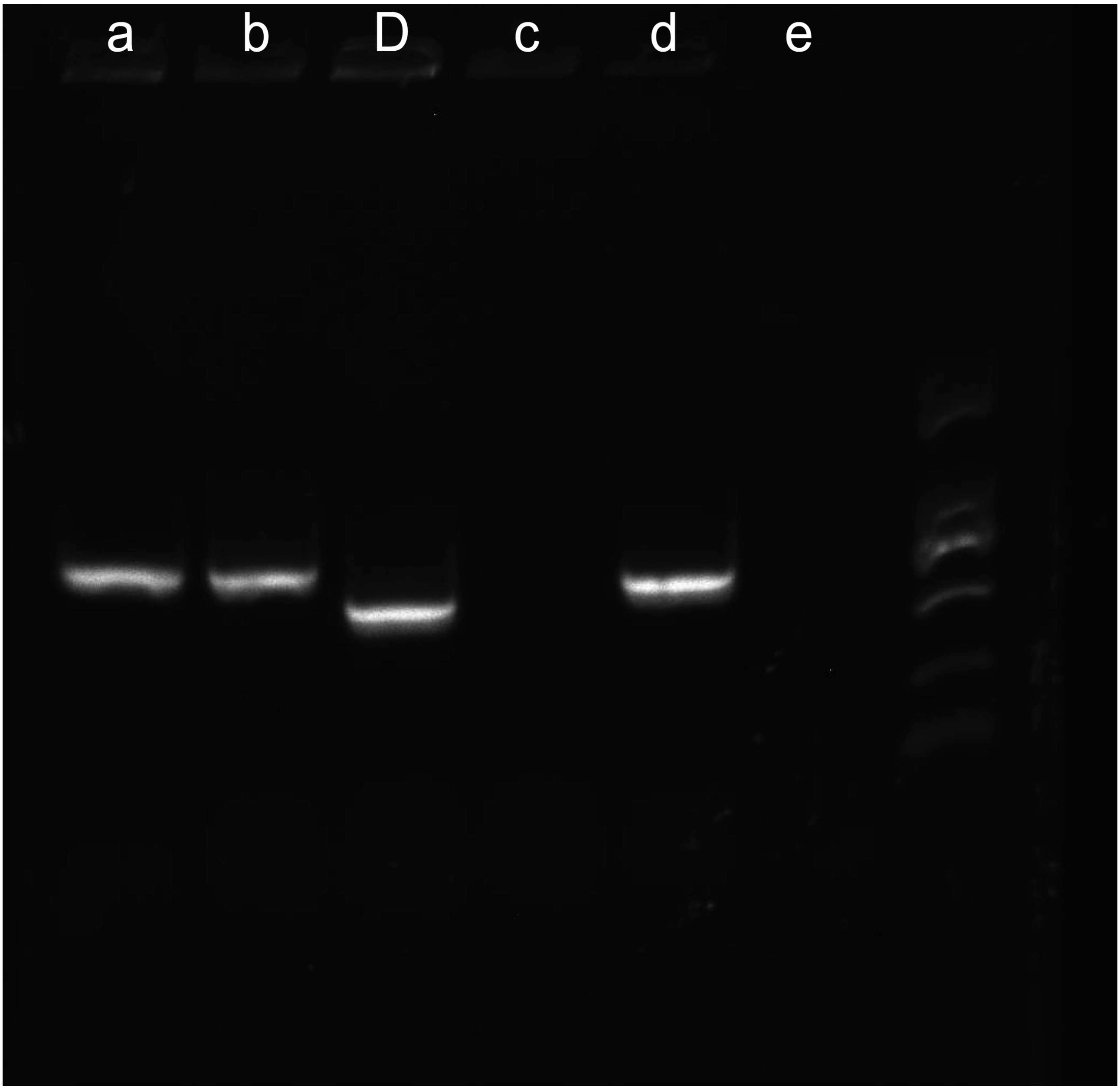

Figure S1 F-1. PCR analysis of the transcription of the *MAT1-1-1* genes in different deletion lines. D-DNA template of WT. **a**. WT (Wild-type *U. botrytis*). **b.** CK is an empty vector transformant. **c.** *ΔmatUbMAT-1,* G418 was used to detect transgene insertion. **d.** *ΔmatUbMAT-2, hygB* was used to detect transgene insertion. **e.** *DmUbMAT-1*, *hygB* and G418 were individually used to detect transgene insertion. Each experiment was repeated at least three independently times.

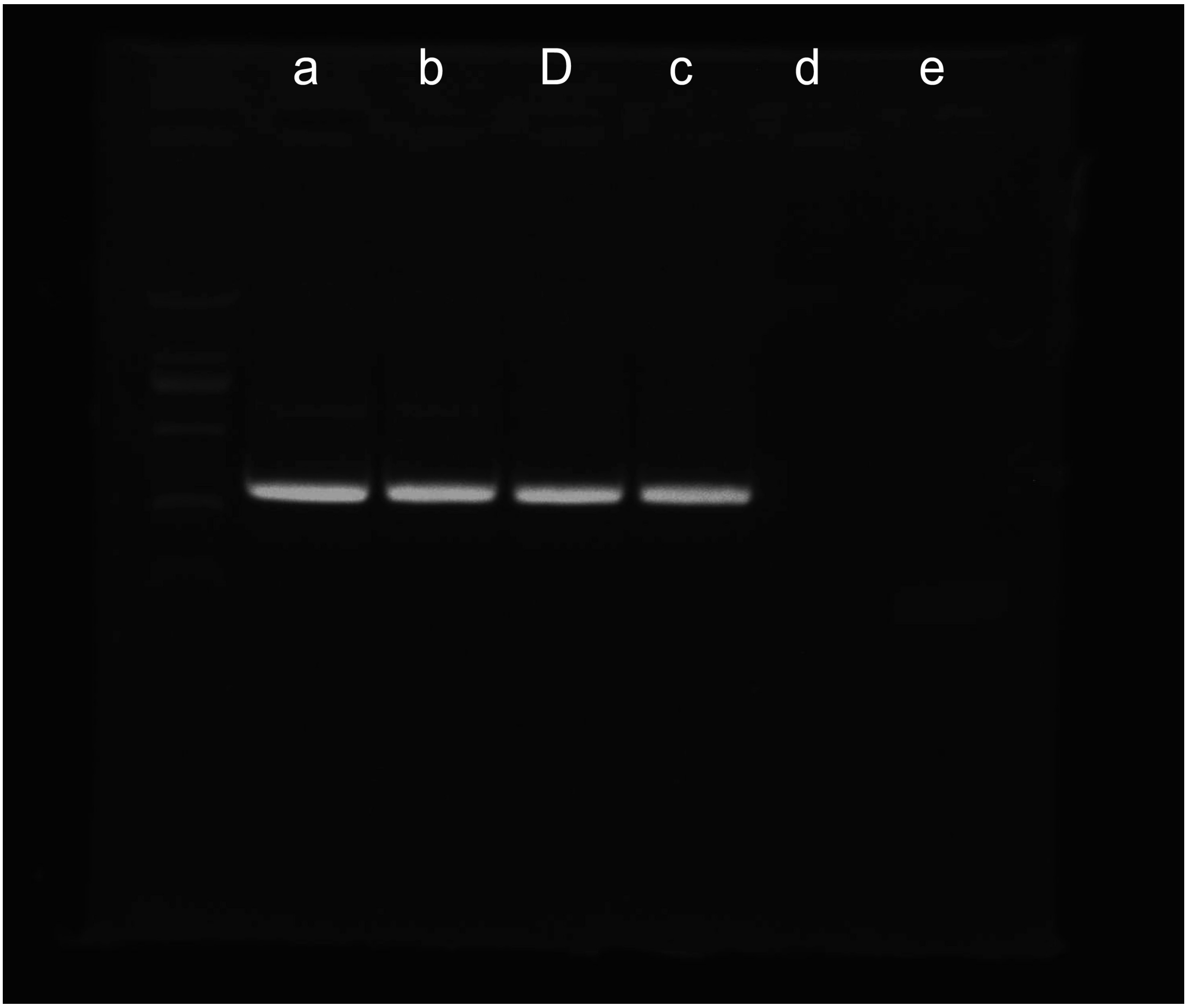

Figure S1 F-2. PCR analysis of the transcription of the *MAT1-2-1* genes in different deletion lines. D-DNA template of WT. **a**. WT (Wild-type *U. botrytis*). **b.** CK is an empty vector transformant. **c.** *ΔmatUbMAT-1,* G418 was used to detect transgene insertion. **d.** *ΔmatUbMAT-2, hygB* was used to detect transgene insertion. **e.** *DmUbMAT-1*, *hygB* and G418 were individually used to detect transgene insertion. Each experiment was repeated at least three independently times.

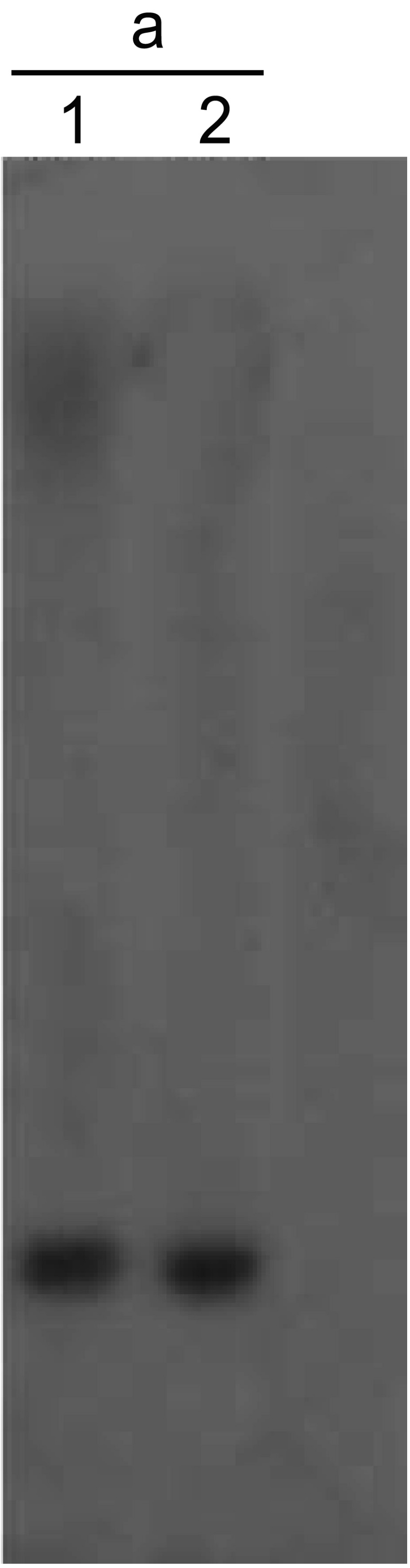

Figure S1 G-1. Northern blot analysis. Twenty micrograms of RNA isolated from WT strain (Wild-type *U. botrytis*). a: 1 and 2 lanes were a 5.8S rRNA-specific probe respectively. Each experiment was repeated at least three independently times.

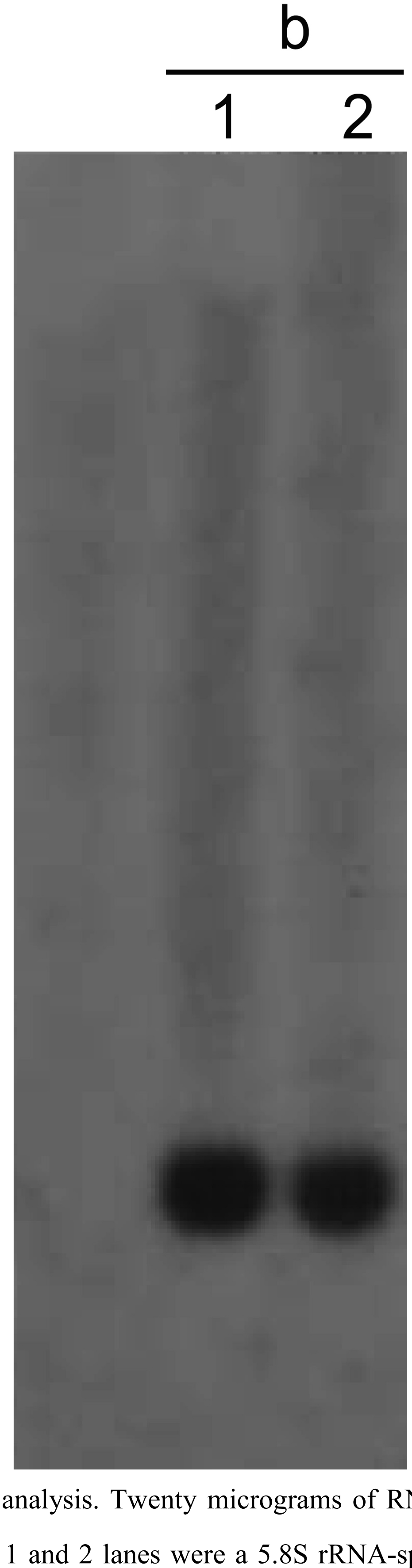

Figure S1 G-2. Northern blot analysis. Twenty micrograms of RNA isolated from CK strain (an empty vector transformant). b: 1 and 2 lanes were a 5.8S rRNA-specific probe respectively. Each experiment was repeated at least three independently times.

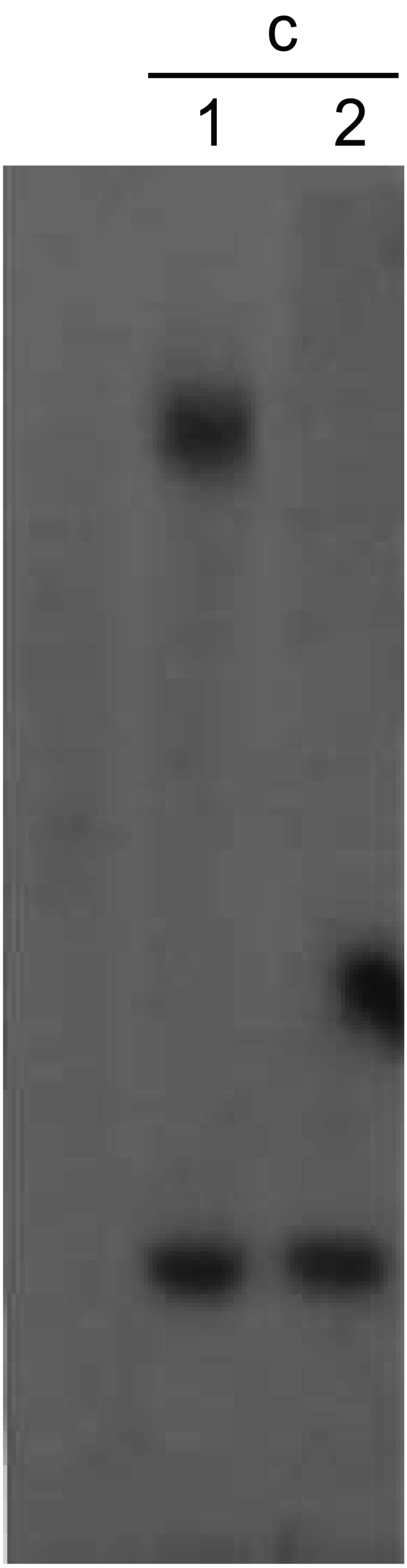

Figure S1 G-3. Northern blot analysis. Twenty micrograms of RNA isolated from *ΔmatUbMAT-1* strain. c: 1 and 2 lanes were a 5.8S rRNA-specific probe respectively. Each experiment was repeated at least three independently times.

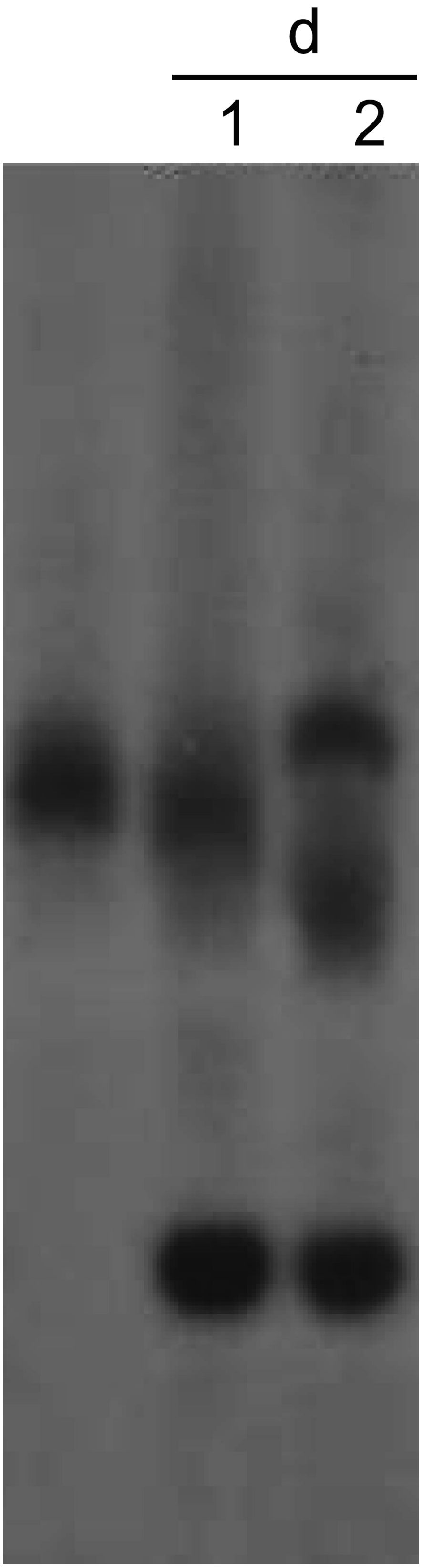

Figure S1 G-4. Northern blot analysis. Twenty micrograms of RNA isolated from *ΔmatUbMAT-2* strain. d: 1 and 2 lanes were a 5.8S rRNA-specific probe respectively. Each experiment was repeated at least three independently times.

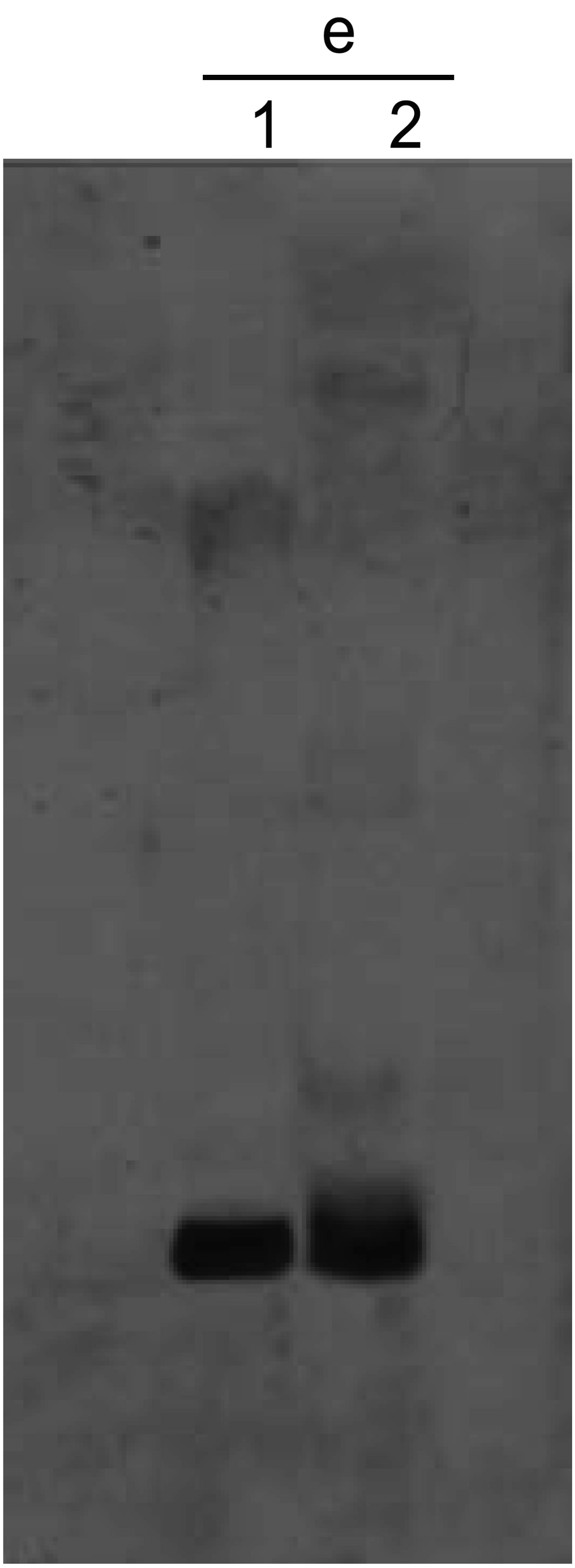

Figure S1 G-5. Northern blot analysis. Twenty micrograms of RNA isolated from *DmUbMAT-1* strain. e: 1 and 2 lanes were a 5.8S rRNA-specific probe respectively. Each experiment was repeated at least three independently times.

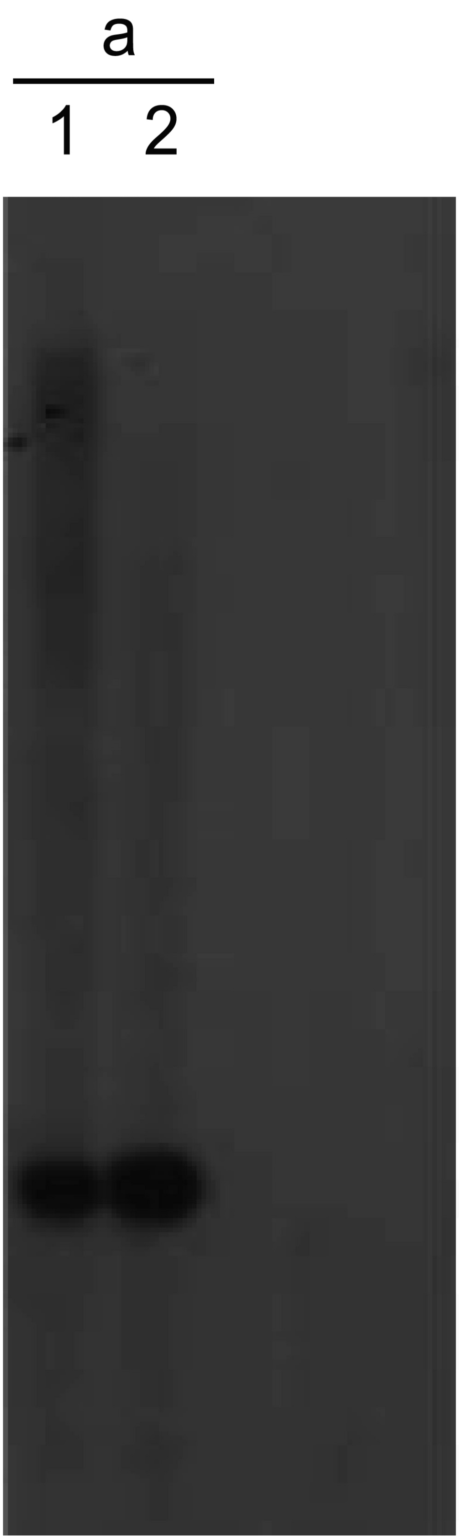

Figure S1 G-a. Northern blot analysis. Twenty micrograms of RNA isolated from WT strain (Wild-type *U. botrytis*). The Northern blot was probed using *MAT1-1-1* and *MAT1-2-1* gene-specific probes in a: 1 and 2 lines. Each experiment was repeated at least three independently times.

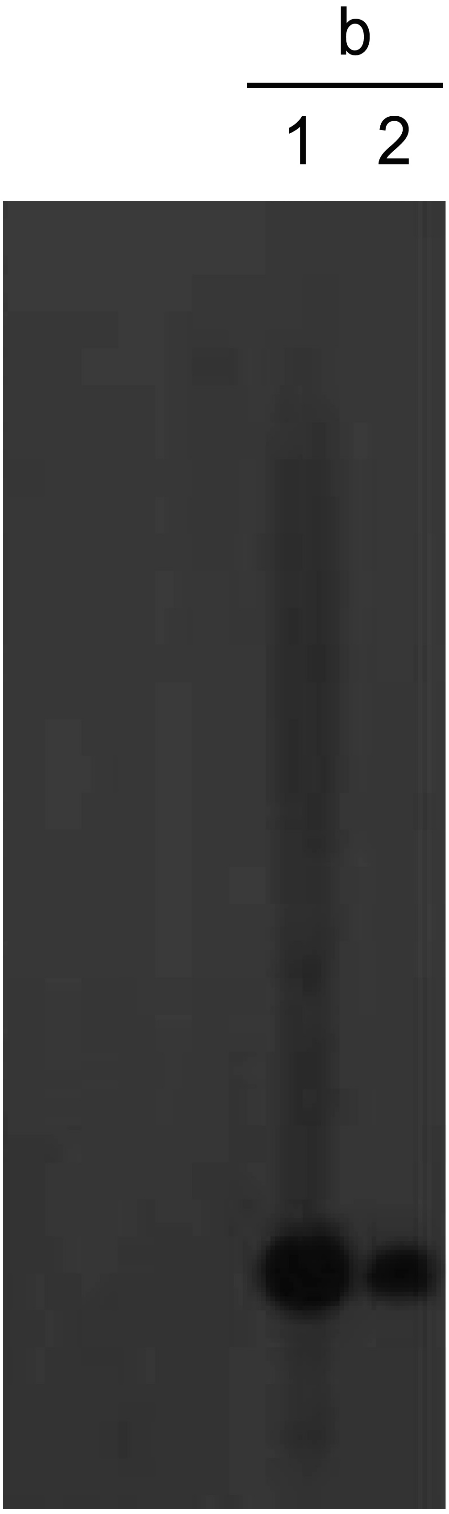

Figure S1 G-b. Northern blot analysis. Twenty micrograms of RNA isolated from CK strain (an empty vector transformant). The Northern blot was probed using *MAT1-1-1* and *MAT1-2-1* gene-specific probes in b: 1 and 2 lines. Each experiment was repeated at least three independently times.

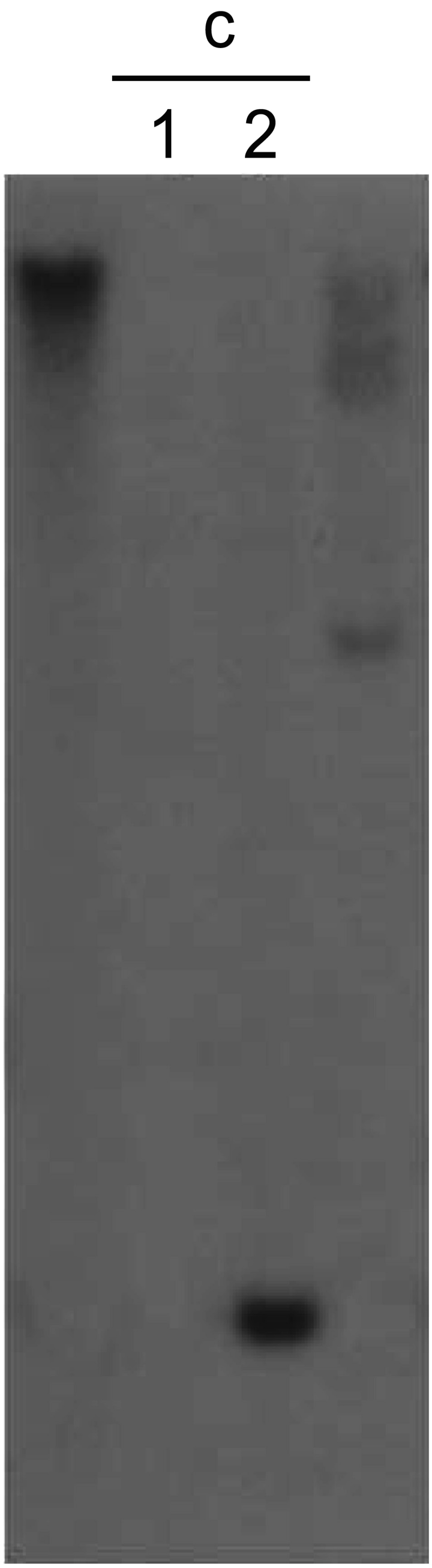

Figure S1 G-c. Northern blot analysis. Twenty micrograms of RNA isolated from *ΔmatUbMAT-1* strain. The Northern blot was probed using *MAT1-2-1* gene-specific probe in c: 1 and 2 lines. Each experiment was repeated at least three independently times.

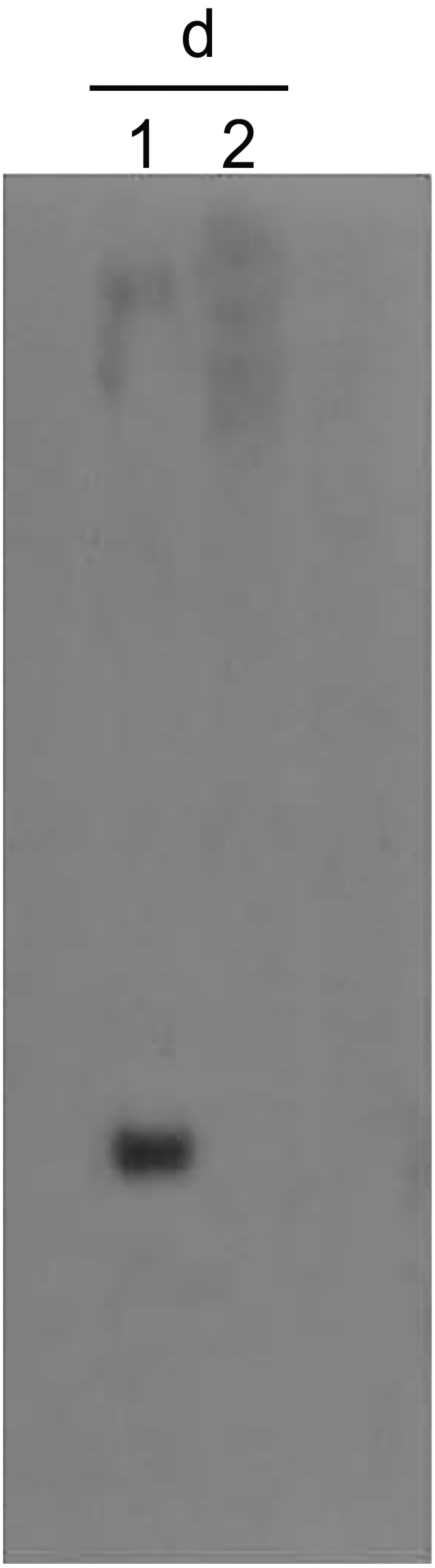

Figure S1 G-d. Northern blot analysis. Twenty micrograms of RNA isolated from *ΔmatUbMAT-2* strain. The Northern blot was probed using *MAT1-1-1* gene-specific probe in d: 1 and 2 lines. Each experiment was repeated at least three independently times.

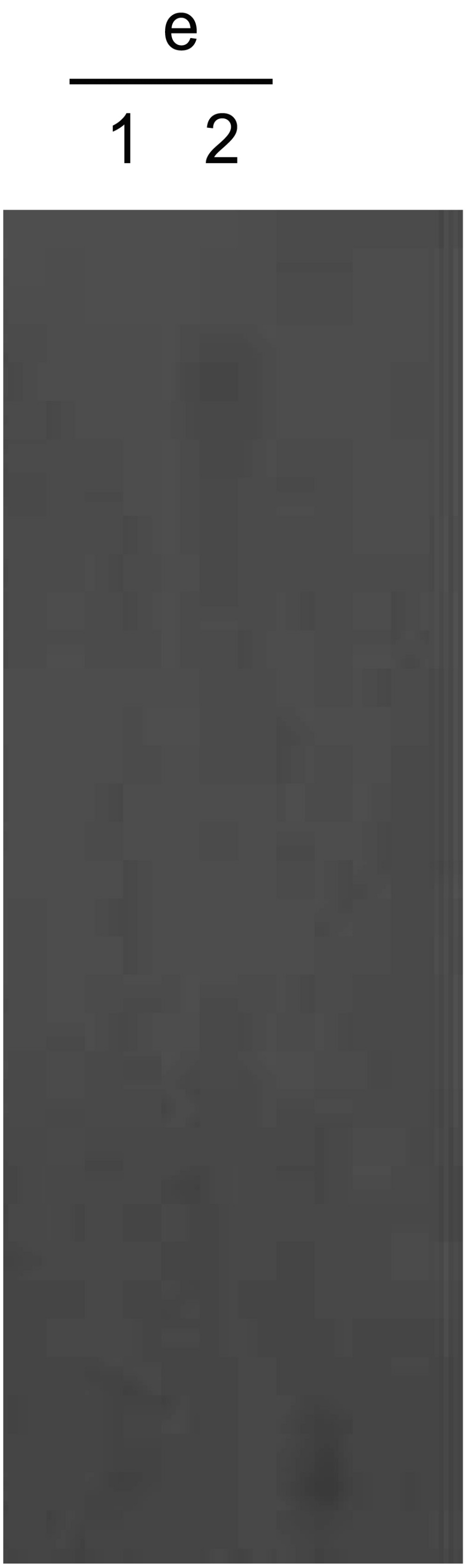

Figure S1 G-e. Northern blot analysis. Twenty micrograms of RNA isolated from *DmUbMAT-1* strain. The Northern blot was probed using *MAT1-1-1* and *MAT1-2-1* gene-specific probe in e: 1 and 2 lines. Each experiment was repeated at least three independently times.

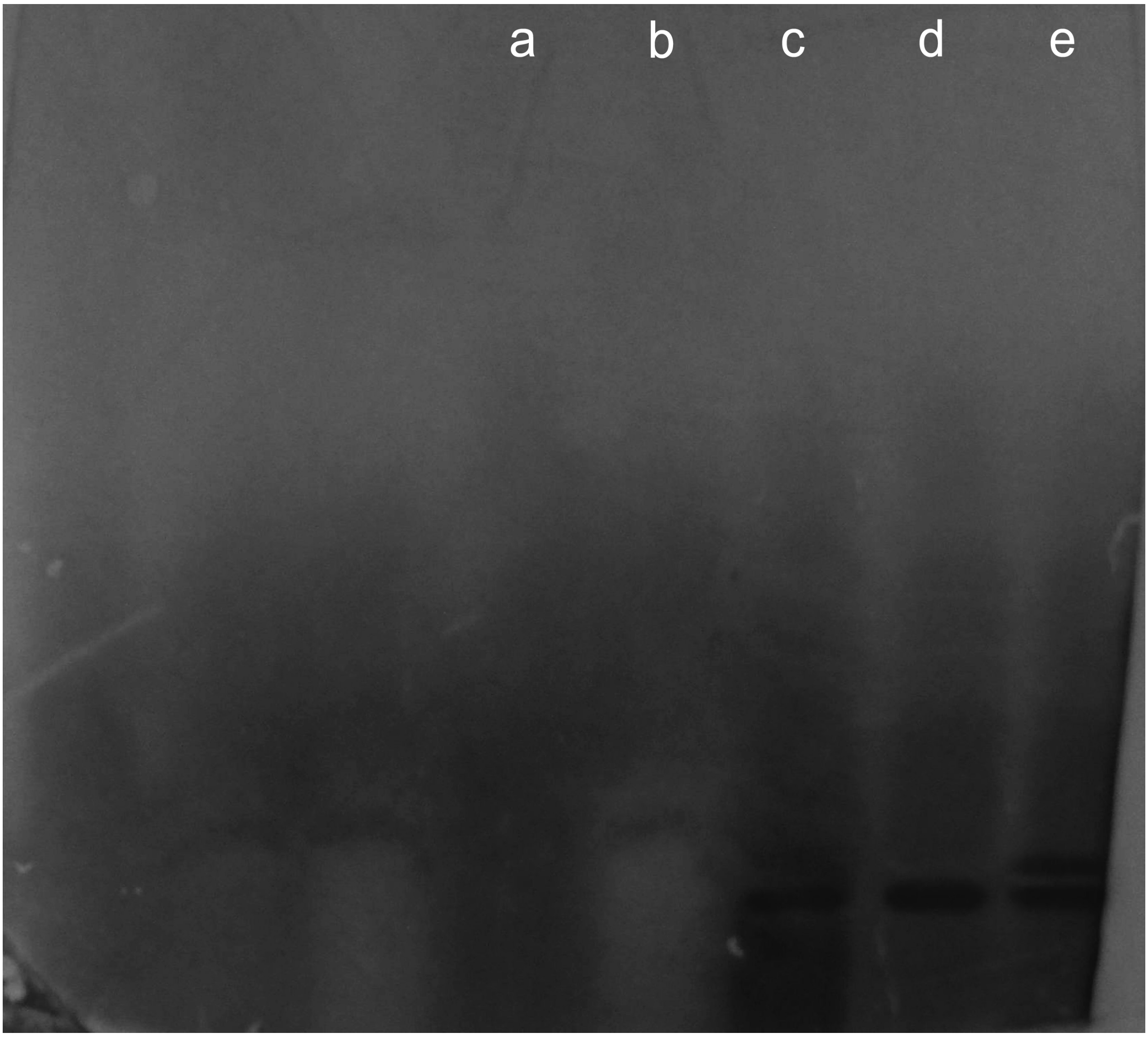

Figure S1 H. For Southern blot analysis, both *hygB* and G418 specific probes were used to detect transgene insertion. WT and CK have no *hygB* and G418 specific insertion. **a**. WT (Wild-type *U. botrytis*). **b.** CK is an empty vector transformant. **c.** *ΔmatUbMAT-1,* G418 was used to detect transgene insertion. **d.** *ΔmatUbMAT-2, hygB* was used to detect transgene insertion. **e.** *DmUbMAT-1*, *hygB* and G418 were individually used to detect transgene insertion. Each experiment was repeated at least three independently times.

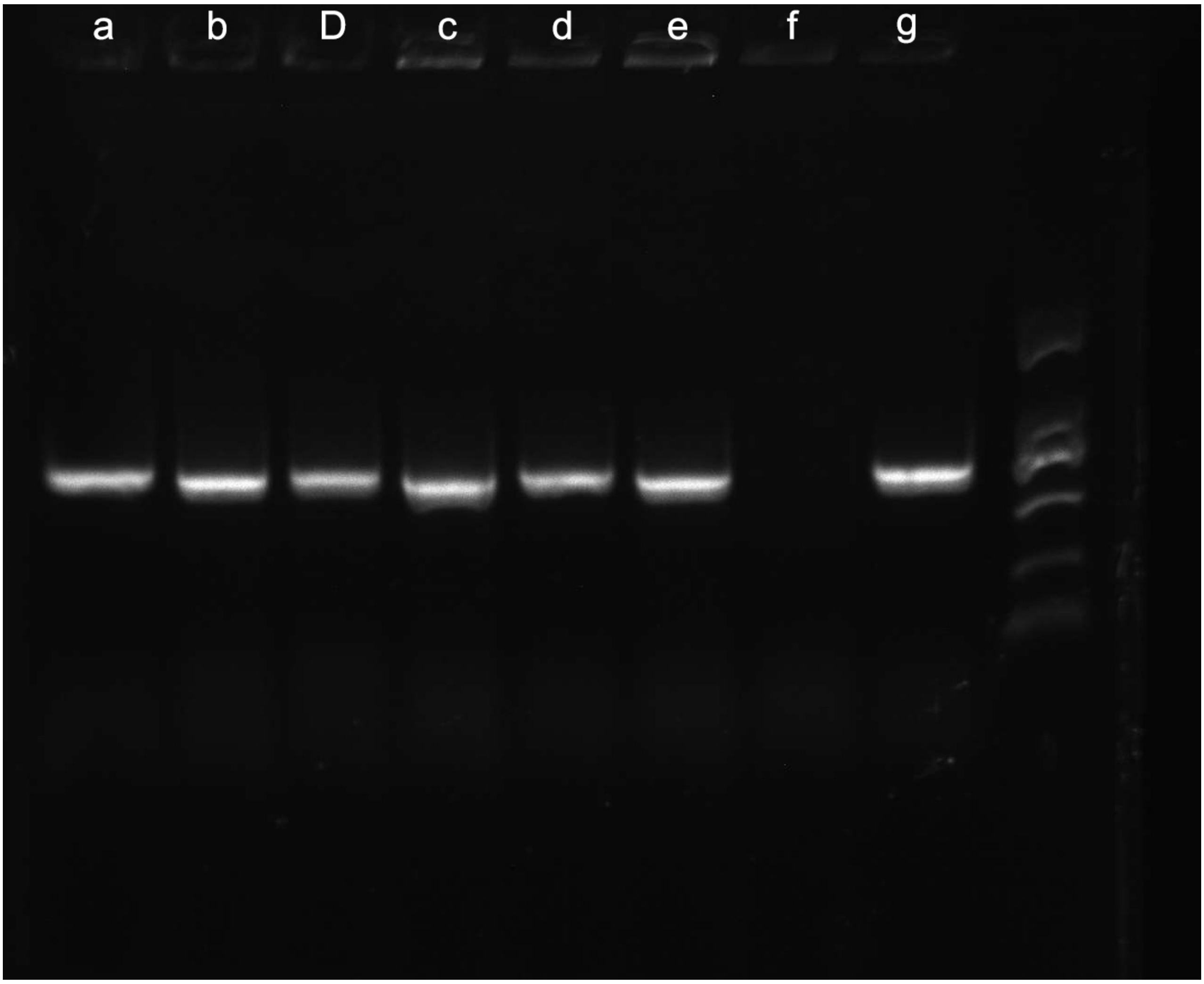

Figure S2 F-1. PCR analysis of *MAT1-1-1* gene transcription in different transgenic lines. D-DNA template of WT. **a.** WT (Wild-type *U. botrytis*), **b.** CK is an empty vector transformant. **c.** *ΔmatUbMAT-1{ChMAT}-1*, **d.** *ΔmatUbMAT-2{ChMAT}-1*, **e**. *DmUbMAT-1{ChMAT}-1-1*, **f.** *DmUbMAT-1{ChMAT}-2-1*, **g.** *DmUbMAT-1{ChMAT}-3-1*. Each experiment was repeated at least three times.

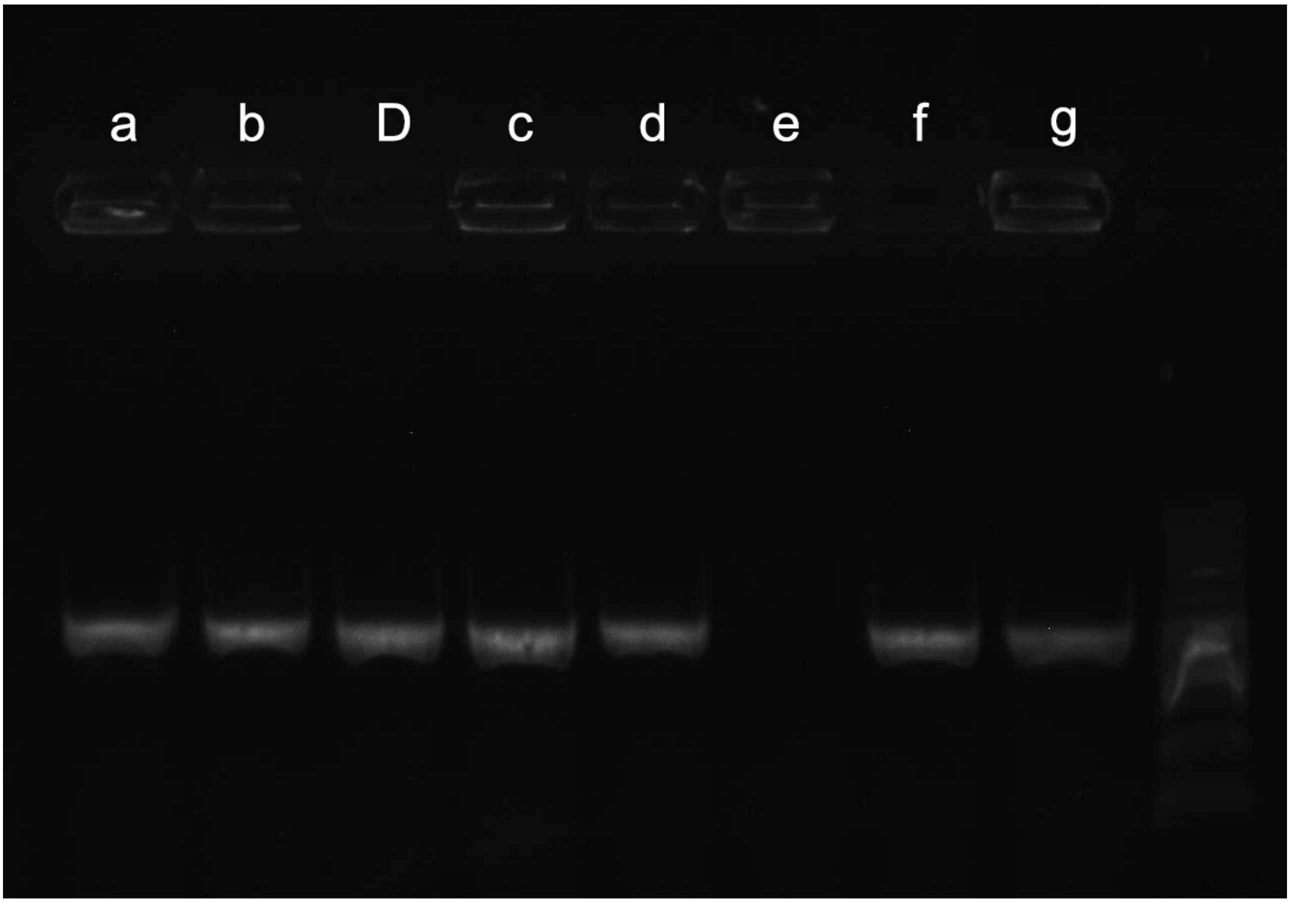

Figure S2 F-2. PCR analysis of *MAT1-1-1* gene transcription in different transgenic lines. D-DNA template of WT. **a.** WT (Wild-type *U. botrytis*), **b.** CK is an empty vector transformant. **c.** *ΔmatUbMAT-1{ChMAT}-1*, **d.** *ΔmatUbMAT-2{ChMAT}-1*, **e**. *DmUbMAT-1{ChMAT}-1-1*, **f.** *DmUbMAT-1{ChMAT}-2-1*, **g.** *DmUbMAT-1{ChMAT}-3-1*. Each experiment was repeated at least three times.

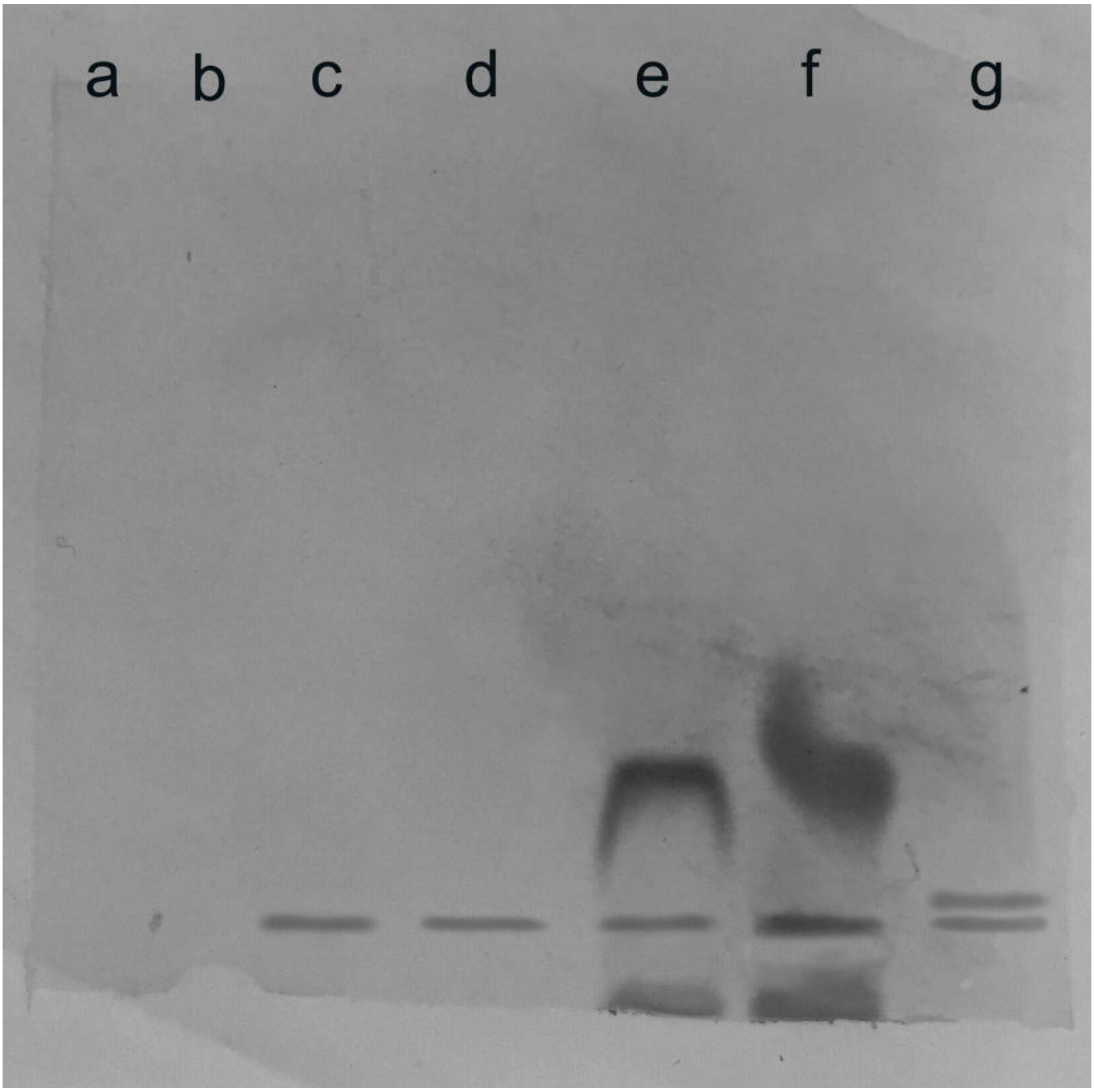

Figure S2 H. For Southern blot analysis, both *hygB* and G418 specific probes were used to detect transgene insertion as shown in Table S1. WT and CK have no *hygB* and G418 specific insertion. **a.** WT (Wild-type *U. botrytis*), **b.** CK is an empty vector transformant. **c.** *ΔmatUbMAT-1{ChMAT}-1*, **d.** *ΔmatUbMAT-2{ChMAT}-1*, **e**. *DmUbMAT-1{ChMAT}-1-1*, **f.** *DmUbMAT-1{ChMAT}-2-1*, **g.** *DmUbMAT-1{ChMAT}-3-1*. Each experiment was repeated at least three times.

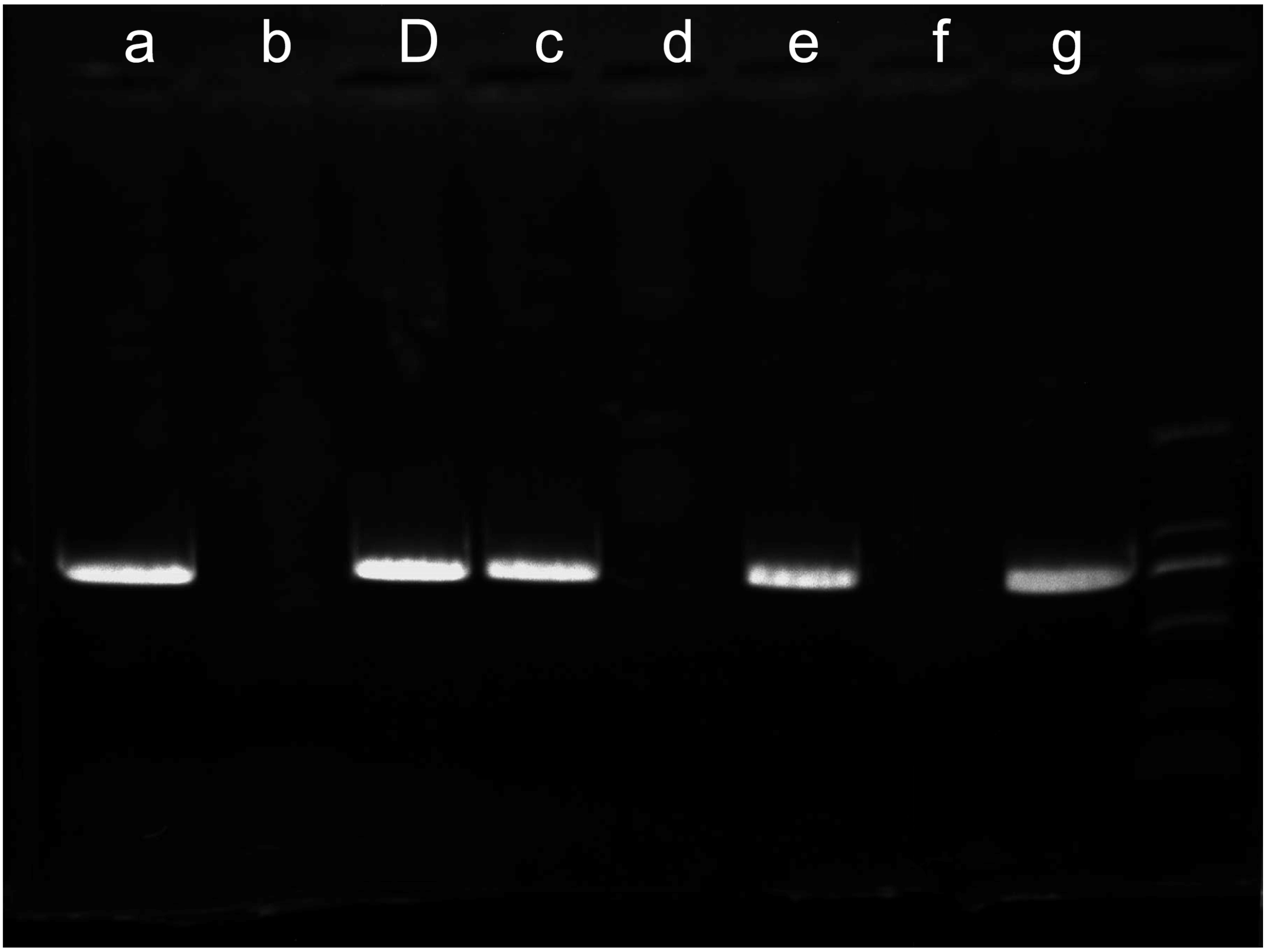

Figure S3 E-1. RT-PCR analysis of the transcription of *MAT1-1-1* genes in different transgenic lines. D-DNA template of WT. **a.** WT is *C. heterostrophus* (2847). **b.** WT1 is *C. heterostrophus* C4-41.7 (MAT-0). **c.** WT2 is *C. heterostrophus* C5 (2829). **d.** WT3 is *C. heterostrophus* C4 (2849). **e.** *ChΔmat0 {UbMAT}-2*. **f.** *ChΔmat0 {UbMAT}-3*. **g.** *ChΔmat0 {UbMAT}-4*. Each experiment was repeated at least three times.

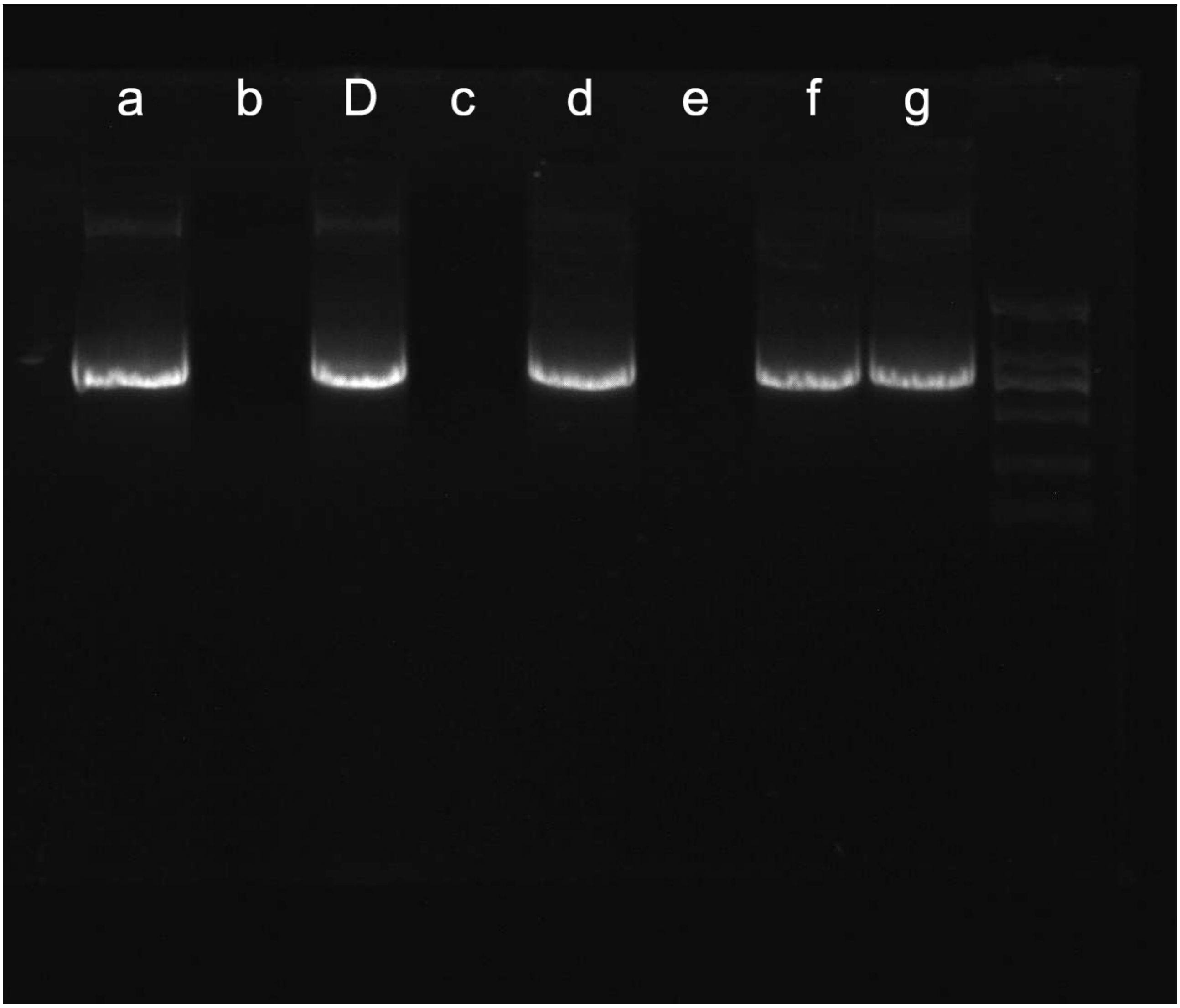

Figure S3 E-2. RT-PCR analysis of the transcription of *MAT1-2-1* genes in different transgenic lines. D-DNA template of WT. **a.** WT is *C. heterostrophus* (2847). **b.** WT1 is *C. heterostrophus* C4-41.7 (MAT-0). **c.** WT2 is *C. heterostrophus* C5 (2829). **d.** WT3 is *C. heterostrophus* C4 (2849). **e.** *ChΔmat0 {UbMAT}-2*. **f.** *ChΔmat0 {UbMAT}-3*. **g.** *ChΔmat0 {UbMAT}-4*. Each experiment was repeated at least three times.

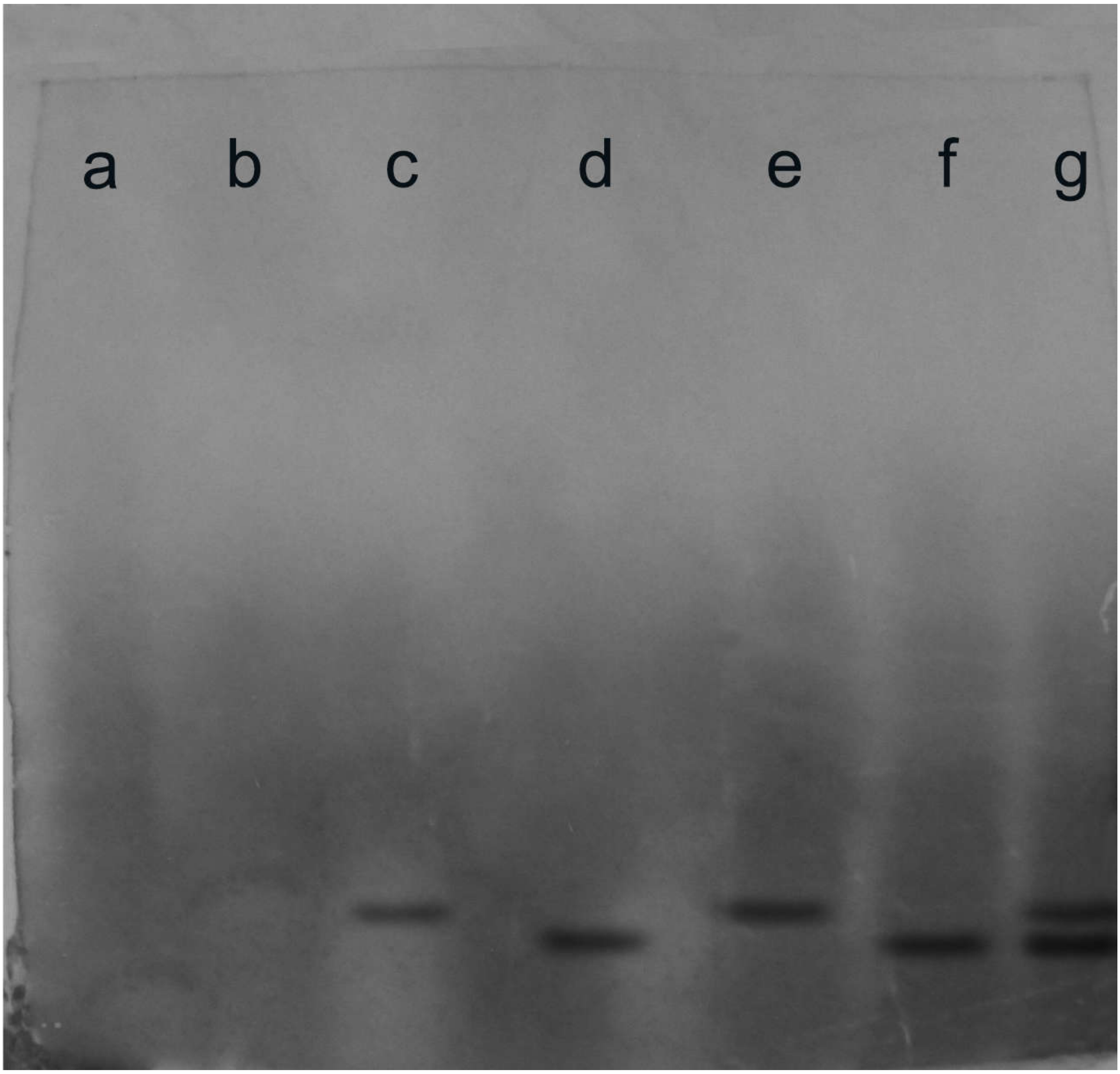

Figure S3 G. For Southern blot analysis, both *hygB* and G418 specific probes were used to detect transgene insertion as shown in Table S1. WT and WT1 have no *hygB* and G418 specific insertion. **a.** WT is *C. heterostrophus* (2847). **b.** WT1 is *C. heterostrophus* C4-41.7 (MAT-0). **c.** WT2 is *C. heterostrophus* C5 (2829). **d.** WT3 is *C. heterostrophus* C4 (2849). **e.** *ChΔmat0 {UbMAT}-2*. **f.** *ChΔmat0 {UbMAT}-3*. **g.** *ChΔmat0 {UbMAT}-4*. Each experiment was repeated at least three times.
